# A Multi‐Functional Nanoadjuvant Coupling Manganese with Toll‐Like 9 Agonist Stimulates Potent Innate and Adaptive Anti‐Tumor Immunity

**DOI:** 10.1002/advs.202402678

**Published:** 2024-09-11

**Authors:** Zhongjie Liu, Shu Li, Yang Xiao, Xiaoyang Liu, Bin Zhang, Qin Zeng, Qiang Ao, Xingdong Zhang

**Affiliations:** ^1^ College of Biomedical Engineering Sichuan University Chengdu 610064 China; ^2^ NMPA Key Laboratory for Quality Research and Control of Tissue Regenerative Biomaterial & Institute of Regulatory Science for Medical Device & National Engineering Research Center for Biomaterials Sichuan University Chengdu Sichuan 610064 China; ^3^ Orthopedic Research Institution, Department of Orthopedics West China Hospital, Sichuan University Chengdu 610041 China

**Keywords:** manganese, nanoadjuvant, TLR9 agonist, tumor immunotherapy, vaccine

## Abstract

The effectiveness of Toll‐like 9 agonists (CpG) as an adjuvant for tumor immunotherapy is restricted due to their insufficient ability to activate anti‐tumor immunity. To address that, the common nutrient metal ions are explored (Mn^2+^, Cu^2+^, Ca^2+^, Mg^2+^, Zn^2+^, Fe^3+^, and Al^3+^), identifying Mn^2+^ as a key enhancer of CpG to mediate immune activation by augmenting the STING‐NF‐κB pathway. Mn^2+^ and CpG are then self‐assembled with epigallocatechin gallate (EGCG) into a nanoadjuvant MPN/CpG. Local delivery of MPN/CpG effectively inhibits tumor growth in a B16 melanoma‐bearing mouse model, reshaping the tumor microenvironment (TME) by repolarizing M2‐type tumor‐associated macrophages (TAMs) to an M1‐type and boosting intra‐tumoral infiltration of CD8^+^/CD4^+^ T lymphocytes and DCs. Furthermore, compared to free CpG, MPN/CpG exhibits heightened accumulation in lymph nodes, enhancing CpG uptake and DC activation, consequently inducing significant antigen‐specific cytotoxic CD8^+^ T cell immune response and humoral immunity. In a prophylactic tumor‐bearing mouse model, MPN/CpG vaccination with OVA antigen significantly delays B16‐OVA melanoma growth and extends mouse survival. These findings underscore the potential of MPN/CpG as a multifunctional adjuvant platform to drive powerful innate and adaptive immunity and regulate TME against tumors.

## Graphical abstract

The effectiveness of CpG as an immune adjuvant for tumor immunotherapy is enhanced by the addition of Mn^2+^ through the activation of the STING‐NF‐κB pathway. Then, the nanoadjuvant MPN/CpG is formed by the self‐assembly of Mn^2+^ and CpG with the coordination of epigallocatechin gallate (EGCG)and significantly inhibits tumor growth and prolongs the animal survival in a B16 melanoma model. The tumor microenvironment is reshaped by MPN/CpG with increased T cell and dendritic cell (DC) infiltration, enhanced antigen‐specific adaptive immune responses.

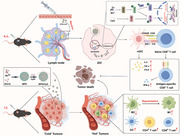

## 1 Introduction

Cancer immunotherapy, which involves activating the body's immune system to suppress the growth and metastasis of tumors, is one of the most effective approaches in cancer treatment.^[^ ^1^ ^]^ In particular, tumor vaccines have the capability to induce tumor‐specific and long‐lasting memory immune responses, thereby inhibiting tumor growth, metastasis, and recurrence.^[^ ^2^, ^3^, ^4^ ^]^ However, the limited immunostimulation by antigen‐presenting cells (APCs) significantly restricts the anti‐tumor efficacy of tumor vaccines.^[^ ^5^ ^]^ Additionally, the immunosuppressive tumor microenvironment poses further challenges to their effectiveness.^[^ ^6^ ^]^ The tumor microenvironment is a complex system conducive to tumor formation, comprising stromal cells, fibroblasts, endothelial cells, innate immune cells, and adaptive immune cells.^[^ ^7^ ^]^ Tumor‐associated macrophages (TAMs), myeloid‐derived suppressor cells (MDSCs), and regulatory T cells (Treg) are the main inhibitory immune cells, with TAMs being the most abundant, constituting up to 50% of the tumor mass.^[^ ^8^ ^]^ TAMs in the TME are mostly of the M2 type, capable of expressing high levels of immune checkpoint ligands^[^ ^9^ ^]^ or recruiting regulatory T cells to suppress CD8^+^ T cell activity.^[^ ^10^ ^]^ Therefore, an increasing number of cancer vaccines are incorporating immune adjuvants to address this deficiency. Adjuvants were first discovered in 1926 and approved for use in diphtheria vaccines in 1932.^[^ ^11^ ^]^ Since then, an increasing number of adjuvants have been developed and brought to market. For instance, lipid‐based adjuvants such as AS01‐AS015 (GSK) have been employed in candidate vaccine formulations targeting conditions such as herpes zoster, malaria, and melanoma.^[^ ^12^ ^]^ Emulsion adjuvants like MF59 (Novartis), AS02, AS04 (GSK), and AF03 (Sanofi Pasteur) have also been successively introduced to the market.^[^ ^13^ ^]^ Recently, the focus of adjuvant development has shifted towards agonists of innate immune system receptors, especially Toll‐like receptor (TLR) agonists.^[^ ^14^ ^]^ These immune adjuvants can stimulate the maturation of innate immune cells, enhance antigen presentation, and subsequently activate T cells by activating the pattern recognition receptor (PRR) signaling pathways.^[^ ^15^, ^16^, ^17^ ^]^ Adjuvants with these immunoenhancing effects are crucial for improving the efficacy of cancer vaccines.

CpG, an unmethylated oligodeoxynucleotide sequence, has been extensively utilized as a potent adjuvant in cancer immunotherapy.^[^ ^18^ ^]^ Upon binding with Toll‐like receptor 9 (TLR9), a pattern recognition receptor in dendritic cells (DCs), CpG promotes DC maturation. It enhances the uptake and cross‐presentation of tumor antigens and induces the expression of co‐stimulatory molecules (CD80, CD86). This process facilitates T cell activation and proliferation. Mature DCs also secrete various cytokines such as IL‐2, IL‐6, IL‐12, and IFN‐β, collaborating to kill tumor cells.^[^ ^19^ ^]^ Recent studies have shown that CpG can enhance the tumor microenvironment. It transforms the predominantly immunosuppressive tumor microenvironment composed of myeloid‐derived suppressor cells, into a TME rich in lymphocytes.^[^ ^20^ ^]^ Additionally, it shifts tumor‐associated macrophages from an M2 phenotype to an M1 phenotype, enhancing their phagocytic activity and inducing CD8^+^ T cell immune responses.^[^ ^21^ ^]^ Unfortunately, despite extensive preclinical research on CpG in cancer therapy, no CpG derivatives have been approved as first‐line cancer treatments.^[^ ^22^ ^]^ This may be attributed to the fact that single CpG is prone to degradation by nucleases, making it challenging for effective recognition by antigen‐presenting cells (APCs) and thus difficult to trigger a significant cellular immune response.^[^ ^23^ ^]^

Metal elements are essential components of life forms, participating in nearly all fundamental biological processes.^[^ ^24^ ^]^ Increasing evidence suggests that metal ions such as Ca^2+^, K^+^, Fe^2+^, Fe^3+^, Zn^2+^, Mn^2+^, etc., play crucial roles in many key immune processes.^[^ ^25^ ^]^ In particular, recent studies have found that Mn^2+^ plays a crucial role in enhancing cGAS sensitivity and enzymatic activity toward double‐stranded DNA. Additionally, it strengthens the binding affinity between cGAMP and STING, thereby activating the cGAS‐STING pathway to defend against DNA virus intrusion.^[^ ^26^ ^]^ Mn^2+^ is crucial for initiating innate immune responses against tumors, playing a pivotal role in enhancing adaptive immune responses against cancer. Mn^2+^ promotes the maturation of dendritic cells and macrophages, enhances the presentation of tumor‐specific antigens, stimulates the activation of CD8^+^ T cells and NK cells, as well as the secretion of type I interferon, making it a potential adjuvant candidate.^[^ ^27^, ^28^, ^29^ ^]^ Nevertheless, the restricted immune activation and limited accumulation of free Mn^2+^ in secondary lymphoid organs, coupled with potential neurotoxicity, hinder its widespread application in cancer immunotherapy.^[^ ^30^, ^31^ ^]^

**Scheme 1:**
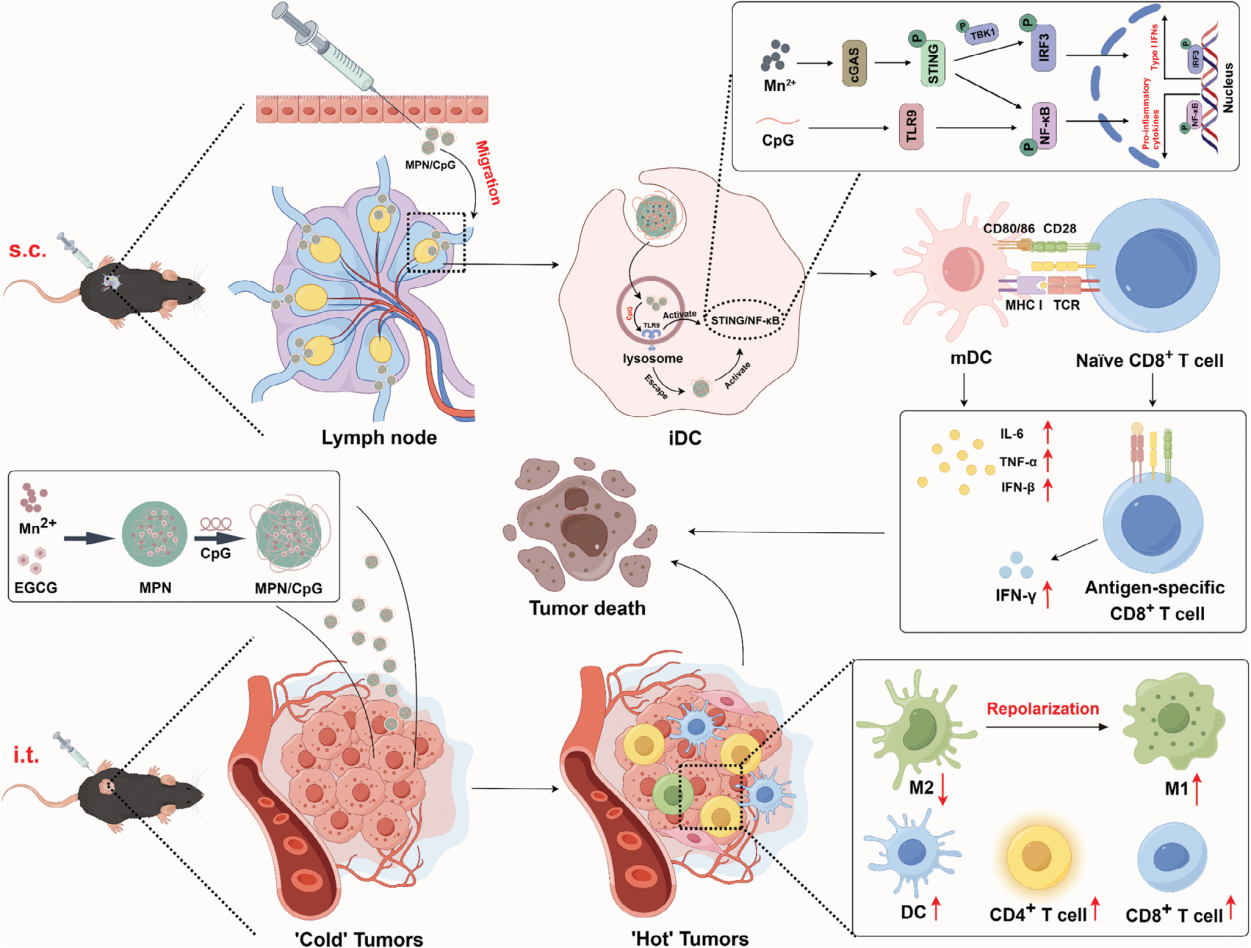
MPN/CpG defends against tumors by activating innate and adaptive immune responses.

Epigallocatechin gallate (EGCG), a naturally non‐toxic tea polyphenol, is rich in catechins and galloyl groups. It can promote tumor cell apoptosis, inhibit tumor cell proliferation, and suppress tumor angiogenesis by modulating signaling pathways and metabolic pathways. Various animal tumor models have confirmed its efficacy in inhibiting the occurrence of multiple cancers, including skin, lung, oral, esophageal, gastric, small intestine, colon, bladder, liver, pancreatic, prostate, and breast.^[^ ^32^ ^]^ Furthermore, due to its ease of binding through covalent or non‐covalent bonds with metal ions, nucleic acids, proteins, polymers, and other substances. EGCG is commonly utilized in the development of nanomaterials.^[^ ^33^ ^]^ Therefore, this study is based on EGCG to construct a dual‐adjuvant nanoplatform that activates multiple signaling pathways for cancer immunotherapy (**Scheme** **1**).

We identified a potential synergistic effect in modulating immune cells between Mn^2+^ and CpG after screening various nutritional metal ions. Their combined use significantly promotes the maturation of dendritic cells, induces cytokine secretion, and stimulates macrophages toward M1‐type polarization. Subsequently, Mn^2+^ and CpG self‐assembled based on EGCG to form a manganese‐polyphenol nanoadjuvant, referred to as MPN/CpG. Intratumoral injection of MPN/CpG reshapes the tumor microenvironment, significantly enhances T lymphocyte infiltration, reverses M2‐type tumor‐associated macrophage polarization, and exhibits a pronounced anti‐tumor effect. Additionally, MPN/CpG can effectively accumulate in lymph nodes and stimulate the maturation of dendritic cells. The tumor vaccine composed of MPN/CpG and OVA demonstrates robust specific cellular immune induction capabilities, effectively preventing the growth of B16‐OVA tumors and extending the survival time of mice.

## 2 Results

### 2.1 Mn^2+^ Synergizes with CpG to Enhance Innate Immune Response

**Figure 1:**
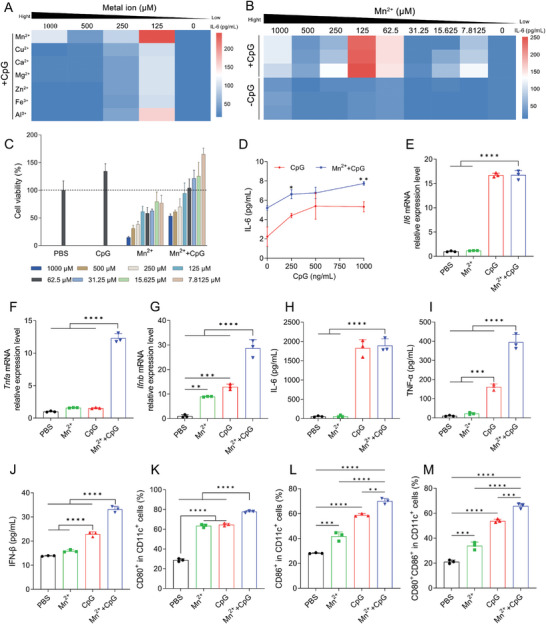
Mn^2+^ synergized with TLR9 agonist CpG to enhance innate immune response. A) Measurement of IL‐6 secretion after RAW264.7 cells were exposed to different concentrations of metal ions in the presence of 500 ng mL^−1^ CpG for 24 hours. Measurement of IL‐6 secretion B) and cell viability C) after RAW264.7 cells received the treatments of different concentrations of Mn^2+^ in the presence or absence of 500 ng mL^−1^ CpG for 24 hours. D) Measurement of IL‐6 secretion after RAW264.7 cells were exposed to 125 µM of Mn^2+^ and different concentrations of CpG for 24 hours. E–G) Quantitative real‐time polymerase chain reaction (qRT‐PCR) was utilized to measure the relative gene expression levels of *Il6*, *Tnfa*, and *Ifnb*. H–J) ELISA detection of IL‐6, TNF‐α, IFN‐β secretion, and K–M) flow cytometry analysis of BMDCs activation phenotypes after BMDCs were exposed to 125 µM of Mn^2+^ and 1 µg mL^−1^ of CpG for 24 hours. Data are presented as means ± SD. Statistical significance was calculated by one‐way ANOVA with Tukey's *post hoc* test. *P < 0.05, **P < 0.01, ***P < 0.001, ****P < 0.0001.

Macrophages are crucial innate immune cells and prevail in an immunosuppressive M2 phenotype in the tumor immune microenvironment. Enhanced immune responsiveness of macrophages to stimuli is critical to screw the phenotype of the cells. We first explored the combined immune‐stimulating effects of different metal ions (Mn^2+^, Cu^2+^, Ca^2+^, Mg^2+^, Zn^2+^, Fe^3+^, and Al^3+^) with the TLR agonist CpG by measuring the secretion of IL‐6. Remarkably, the addition of moderate concentrations of Mn^2+^ and Al^3+^ significantly increased IL‐6 secretion in RAW264.7 cells compared to using CpG alone (**Figure** **1**A). Considering that Mn^2+^ exhibited the most potent effect and has been utilized as an innate immune stimulant in DNA virus infection and immunotherapy,^[^ ^26^ ^]^ we further extended the concentration of Mn^2+^ and observed that only moderate concentrations of Mn^2+^ significantly enhanced IL‐6 secretion, without displaying a concentration‐dependent trend from low to high (Figure 1B). This could be attributed to the pronounced cytotoxicity of high Mn^2+^ concentrations on cells, thus impacting IL‐6 secretion (Figure 1C). Subsequently, with the concentration of Mn^2+^ fixed, we introduced varying concentrations of CpG and observed a concentration‐dependent increase in the secretion of IL‐6. The levels of IL‐6 secretion showed a significant enhancement compared to using CpG alone (Figure 1D). This indicates that the secretion of IL‐6 is influenced by the concentration of CpG. Interestingly, Mn^2+^ demonstrates a synergistic stimulatory effect with other TLR agonists, such as the TLR1/2 agonist Pam3CSK4 and the TLR4 agonist LPS (Figure S1, Supporting Information). This implies that the synergistic interaction between Mn^2+^ and TLR agonists may be universal, suggesting the existence of some underlying regulatory mechanisms. Based on these findings, we chose 125 µM Mn^2+^ and 1 µg mL^−1^ CpG to further investigate their combined immune‐stimulating effects on BMDCs.

To confirm the synergistic enhancement of cytokine secretion by Mn^2+^ and CpG in BMDCs, we evaluated the expression of IL‐6, TNF‐α, and IFN‐β at gene and protein levels using qRT‐PCR and ELISA kits. The results revealed that, while there was no significant change in *Il6* mRNA and IL‐6 expression levels in the Mn^2+^ + CpG group compared to the CpG group (Figure 1E,H), there were strong synergistic effects in the expression of *Tnfa* mRNA, *Ifnb* mRNA (Figure 1F,G), and TNF‐α, IFN‐β (Figure 1I,J) when Mn^2+^ and CpG were used together, whereas the Mn^2+^ and CpG groups showed limited enhancement. Similarly, in the experiment promoting BMDCs maturation, Mn^2+^ + CpG group exhibited the highest level of maturation, showing elevated expression of mature markers CD80 and CD86 (Figure 1K–M). Furthermore, Mn^2+^ can modulate macrophage polarization. The addition of 125 µM Mn^2+^ to macrophages induces polarization from the M0 phenotype to the M1 (CD86^+^) phenotype. When both CpG and Mn^2+^ are treated to the cells, the level of M1 polarization is significantly elevated (Figure S2, Supporting Information). These results collectively suggest that the synergy between Mn^2+^ and CpG effectively modulates innate immune responses, particularly dendritic cell maturation and macrophage polarization.

### 2.2 Mn^2+^ and CpG were Self‐Assembled into Nanoparticles with the Coordination of EGCG

**Figure 2:**
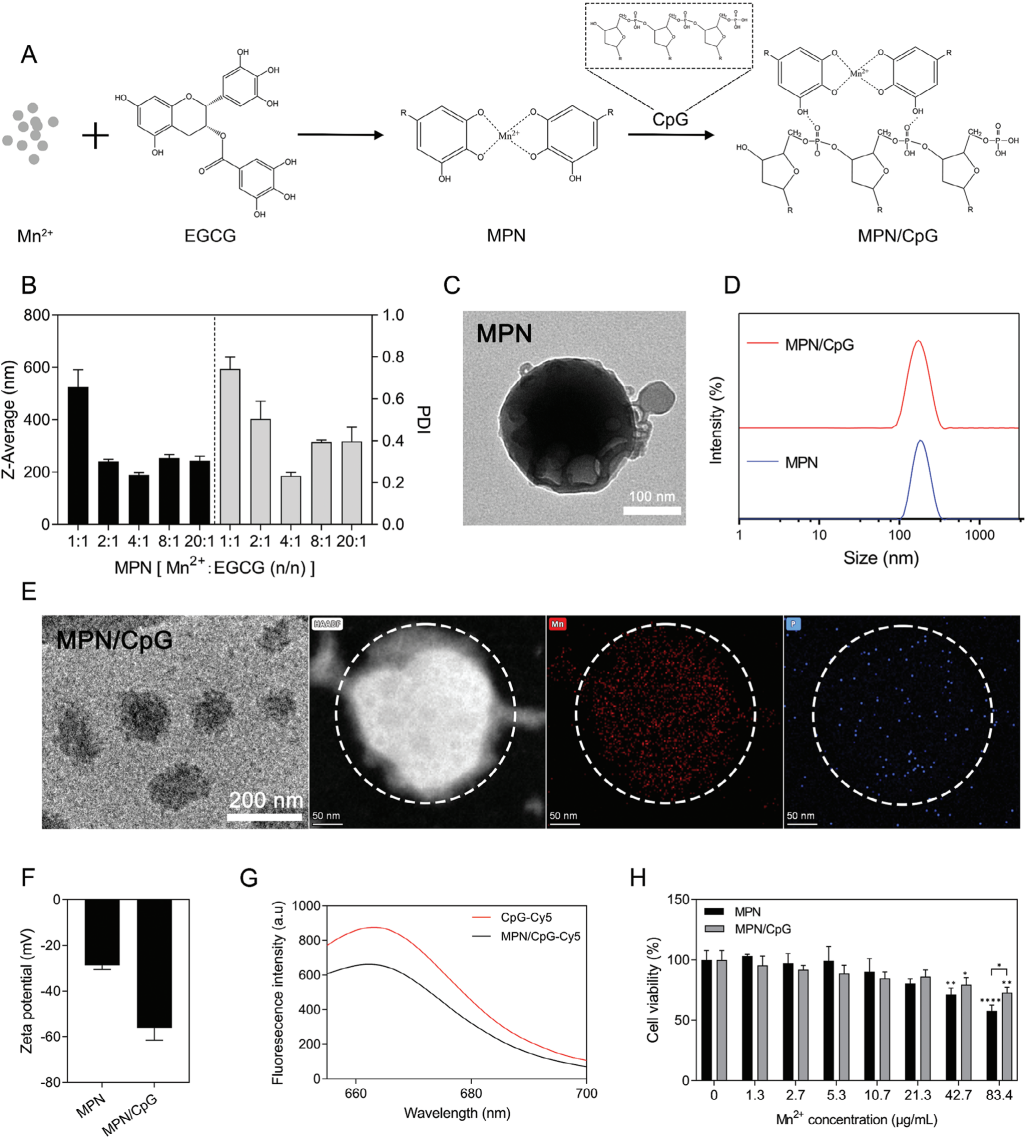
Synthesis, characterization and cytotoxicity of MPN/CpG nanoadjuvant. A) The schematic presentation of MPN/CpG self‐assembly through metal coordination and hydrogen bonding. B) The average particle size of MPN particles composing Mn^2+^ and EGCG at different molar ratios measured by dynamic light scattering (DLS). C) Transmission electron microscope (TEM) image of MPN/CpG at a molar ratio of 4:1 for Mn^2+^ and EGCG, scale bar = 100 nm. DLS analysis of the particle size distribution D) and zeta potential F) of MPN and MPN/CpG. (E) TEM image (scale bar = 200 nm) and HAADF‐STEM image (scale bar = 50 nm) of MPN/CpG. G) Fluorescence spectrophotometer measuring the fluorescence intensity of CpG‐Cy5 and MPN/CpG‐Cy5 at 650 nm to 700 nm. H) Cell viability was assessed using CCK‐8 after RAW264.7 cells were exposed to different concentrations of MPN or MPN/CpG for 24 hours. Data are presented as means ± SD. Significance symbols without additional annotation represent significant comparisons with the control group. Statistical significance was calculated by one‐way ANOVA with Tukey's *post hoc* test. *P < 0.05, **P < 0.01, ****P < 0.0001.

Although Mn^2+^ and CpG exhibit a robust synergistic effect in vitro, their free mixtures may have the limitation of poor co‐delivery during in vivo therapy. To address this issue, we chose epigallocatechin gallate (EGCG) as a carrier to bind Mn^2+^ and CpG through multivalent ligand interactions of phenolic hydroxyl groups and hydrogen bonding (**Figure** **2**A). By varying the molar ratio of Mn^2+^ to EGCG, we obtained MPNs with different particle sizes. Ultimately, we identified the optimal molar ratio as 4:1, resulting in MPNs with the smallest particle size and polydispersity index (PDI). Dynamic light scattering (DLS) measurements revealed a particle size of 189.6 nm ± 6.9 nm and a PDI of 0.23 ± 0.01 nm for MPN (Figure 2B). Transmission Electron Microscopy (TEM) images reveal a spherical structure of MPN (Figure 2C). Subsequently, MPN was mixed with CpG at room temperature to obtain MPN/CpG, exhibiting a narrower size distribution similar to that of MPN (Figure 2D). TEM and HAADF‐STEM images illustrate that MPN/CpG adopts an irregular spherical shape with a diameter of approximately 122 nm, containing manganese (Mn) and phosphorus (P) elements (Figure 2E). Due to the negatively charged CpG payload, the zeta potential of MPN/CpG was significantly lower than that of MPN (−28.8 mV vs −56.1 mV) (Figure 2F). Additionally, the binding of MPN and CpG‐Cy5 led to fluorescence quenching, resulting in reduced fluorescence intensity in MPN/CpG‐Cy5 (Figure 2G). The above research results collectively confirm the successful loading of CpG to MPN. Subsequently, we assessed the cytotoxicity and stability of MPN/CpG. CCK‐8 assay revealed that there was no significant effect on cell viability when the Mn^2+^ concentration was below 21.3 µg mL^−1^. However, when the concentration exceeded 21.3 µg mL^−1^, MPN demonstrated a more pronounced inhibition of cell viability compared to MPC/CpG. (Figure 2H). After 48 hours of storage at 4 °C, there was only a slight change in particle size (Figure S3, Supporting Information), indicating the excellent stability of MPN/CpG.

### 2.3 MPN/CpG Stimulates BMDC Maturation and M1‐Type Macrophage Polarization in Vitro

**Figure 3:**
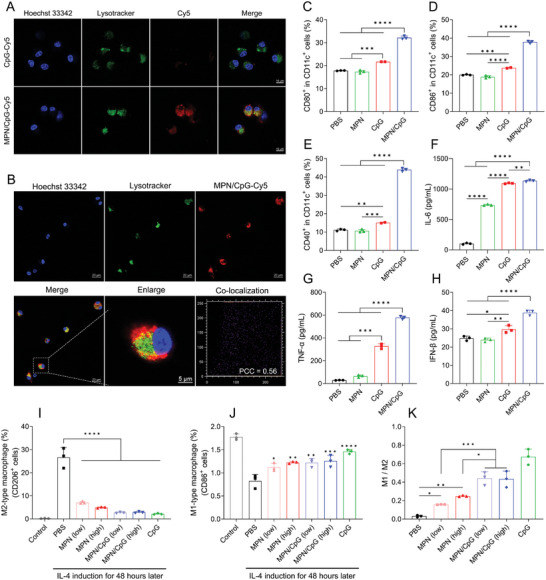
MPN/CpG promoted the uptake of CpG and stimulated the activation of BMDCs in vitro. A) The cellular uptake detected by confocal laser scanning microscope (CLSM) after CpG‐Cy5 or MPN/CpG‐Cy5 were treated to BMDCs for 0.5 hours, scale bar = 10 µm. The cell nucleus and lysosomes were stained with Hoechst 33342 (blue) and Lysotracker (green), respectively. B) The co‐localization of MPN/CpG‐Cy5 (red) and lysosomes (green) after BMDCs were treated with MPN/CpG‐Cy5 for 4 hours, scale bar = 20 µm or 5 µm. The Pearson correlation coefficient (PCC) was calculated using ImageJ. BMDCs were treated with different materials for 24 hours to detect surface marker levels CD80 C), CD86 D), CD40 E) and cytokine secretion levels IL‐6 F), TNF‐α G), IFN‐β H). RAW264.7 cells were induced by IL‐4 for 48 hours and then treated with different materials for 24 hours. M2‐type I) macrophages and M1‐type J) macrophages and their relative proportions K) were detected. Data are presented as means ± SD. Significance symbols without additional annotation represent significant comparisons with the control group. Statistical significance was calculated by one‐way ANOVA with Tukey's *post hoc* test. *P < 0.05, **P < 0.01, ***P < 0.001, ****P < 0.0001.

To evaluate the immunomodulatory response of MPN/CpG as a nanoadjuvant in BMDCs, we initially assessed the cellular uptake of MPN/CpG. After CpG‐Cy5 or MPN/CpG‐Cy5 was treated to BMDCs for 0.5 hours, the localization of CpG was observed by confocal microscopy, the CpG‐Cy5 group exhibited only weak fluorescence fusion signals (**Figure** **3**A), with a Pearson's correlation coefficient (PCC) of 0.47 (Figure S4A, Supporting Information). The weak co‐localization suggests limited uptake of free CpG by BMDCs. In contrast, MPN/CpG‐Cy5 demonstrated more effective cellular uptake, exhibiting a significant overlap between the red fluorescence and the green fluorescence of lysosomes (Figure 3A), resulting in a PCC of 0.69 (Figure S4B, Supporting Information). This strong co‐localization indicates efficient uptake of MPN/CpG by BMDCs. Moreover, MPN/CpG colocalized with TLR9 fluorescence (Figure S5A, Supporting Information), indicating a potential interaction between them. This provided a foundation for the activation of downstream signaling pathways. Upon the co‐incubation time was extended to 4 hours, the overlapped fluorescence signals showed a noticeable separation, with the PCC decreasing from 0.69 to 0.56 (Figure 3B). This suggests that MPN/CpG‐Cy5 began to escape from lysosomes into the cytoplasm. During lysosomal escape, a portion of the MPN/CpG retained its intact morphology and size (Figure S5B, Supporting Information), allowing Mn^2+^ to transfer to the cytoplasm. This transfer was crucial for activating the cytoplasmic STING pathway.

Next, we evaluated the activation of BMDCs after nanoparticle treatment using flow cytometry. Although the expression levels of surface activation markers (CD80, CD86, and CD40) in the CpG group were significantly higher than those in the PBS and MPN groups, the MPN/CpG group still exhibited higher activation levels. Specifically, the expression levels of CD80, CD86, and CD40 were 1.48, 1.59, and 2.91 times higher, respectively, compared to the CpG group (Figure 3C–E). Additionally, we measured the cytokine levels in the supernatant of BMDCs. The MPN/CpG group effectively stimulated the secretion of IL‐6, TNF‐α, and IFN‐β, significantly higher than the levels observed in the PBS, MPN, and CpG groups (Figure 3F–H). Moreover, the gene expression levels followed a similar trend (Figure S6, Supporting Information). These findings indicate that MPN/CpG can effectively stimulate the maturation of BMDCs.

Considering the synergistic regulatory capacity of Mn^2+^ and CpG on macrophage polarization toward M1, we hypothesized that they might also synergistically promote the repolarization of M2‐type macrophages toward M1. To test this hypothesis, macrophages from all experimental groups were initially induced toward M2 polarization with IL‐4. Subsequently, materials with varying Mn^2+^ content were added for a 24‐hour treatment, and the polarization status was assessed by detecting the markers CD86 and CD206 for M1 and M2 macrophages, respectively. In comparison to the negative control group, IL‐4‐induced macrophages expressed higher levels of CD206 and lower levels of CD86, indicative of a predominantly M2 phenotype. However, upon the addition of nanoadjuvants, this situation was significantly reversed. In comparison to the PBS group, the other treatment groups exhibited a significant decrease in the expression level of CD206 (Figure 3I) and a significant increase in that of CD86 (Figure 3J). The ratio of M1 to M2 macrophages also significantly increased, especially in the MPN/CpG group (Figure 3K), suggesting a repolarization of macrophages from M2 to M1.

### 2.4 MPN/CpG Activates the STING‐NF‐κB Pathway in BMDCs

**Figure 4:**
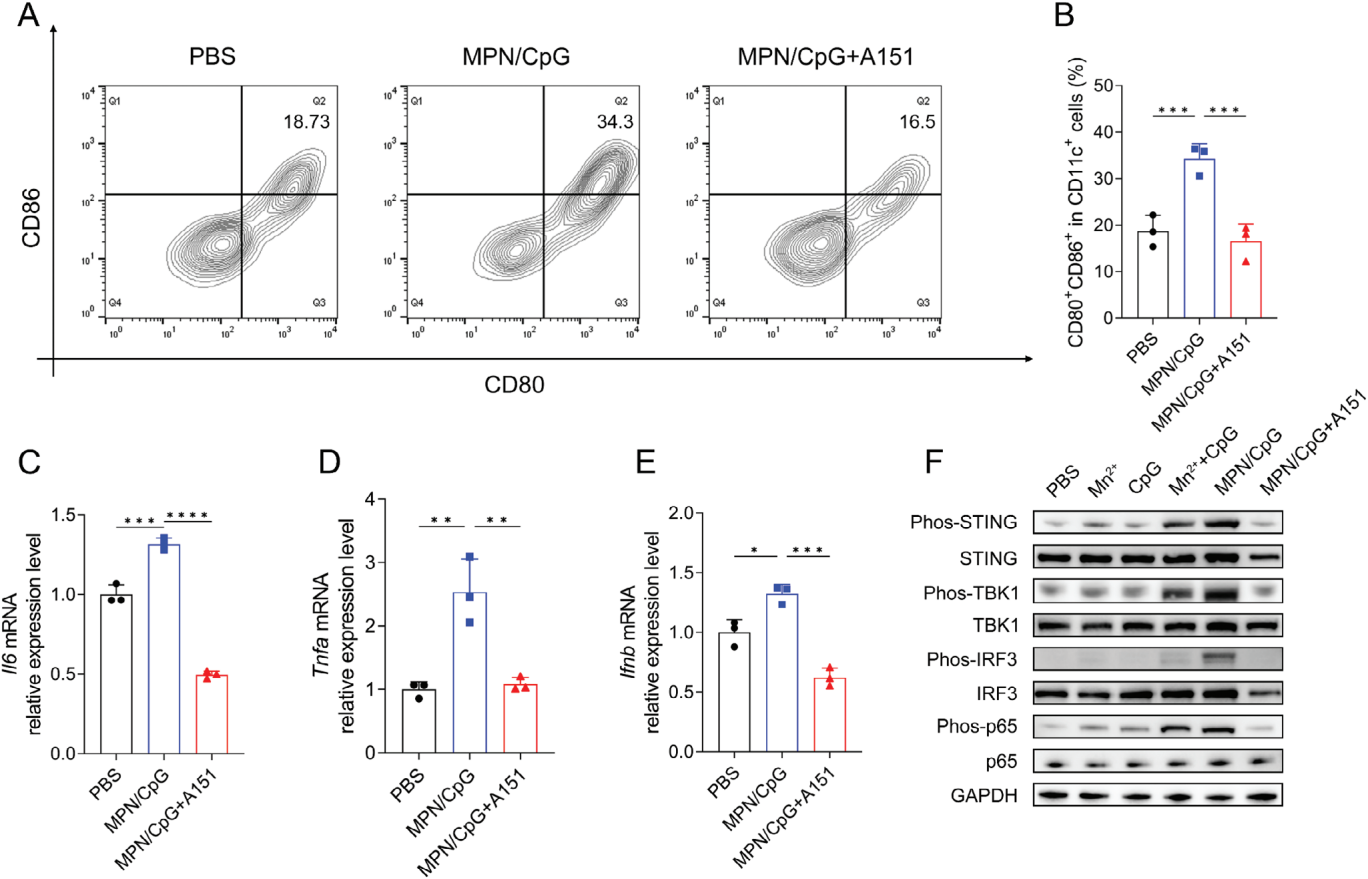
MPN/CpG enhances BMDC maturation by activating the STING‐NF‐κB pathway. After being pre‐treated with or without the A151 inhibitor (1 µM) for 1 hour, BMDCs were exposed to MPN/CpG for 6 hours. A, B) The expression levels of maturation makers on the surface of BMDCs and the mRNA levels of C) *Il6*, D) *Tnfa*, and E) *Ifnb* genes were then assessed. F) The activation levels of the STING‐NF‐κB pathway were assessed by using western blot after BMDCs were exposed to Mn^2+^, CpG, a mixture of Mn^2+^ and CpG, or MPN/CpG nanoparticles for 0.5 hours. In the MPN/CpG + A151 group, the A151 inhibitor (1 µM) was pre‐treated to BMDCs for 1 hour. Data are presented as means ± SD. Statistical significance was calculated by one‐way ANOVA with Tukey's *post hoc* test. *P < 0.05, **P < 0.01, ***P < 0.001, ****P < 0.0001.

Next, we explored the specific mechanism by which MPN/CpG activates BMDCs. A151 (ODN TTAGGG) is an inhibitory oligonucleotide that can suppress the pathways of TLR9 and cGAS‐STING. MPN/CpG promoted the expression of maturation markers CD80 and CD86, but the promotion mediated by MPN/CpG was then completely abolished after a pre‐treatment with A151 for 1 hour (**Figure** **4**A,B). Similarly, the inhibitor also counteracted the promotion effect of MPN/CpG on *Il6*, *Tnfa*, and *Ifnb* gene expression levels (Figure 4C–E).

Subsequently, we assessed the activation of the STING‐NF‐κB pathway by using western blot. The Figure 4F and Figure S7 (Supporting Information) indicated that the combination of Mn^2+^ plus CpG effectively enhanced the protein expression of phosphorylated STING, TBK1, IRF3, and p65 compared to the PBS control, Mn^2+^ or CpG alone treatment, suggestion a synergistical effect between Mn^2+^ and CpG. However, when the A151 inhibitor was added, the activation of STING‐NF‐κB pathway was completely inhibited. These results indicate that the activation of the STING‐NF‐κB pathway is the important molecular mechanism in mediating the synergistical adjuvant effect.

### 2.5 MPN/CpG Inhibits Tumor Growth in a Therapeutic Mouse Model

**Figure 5:**
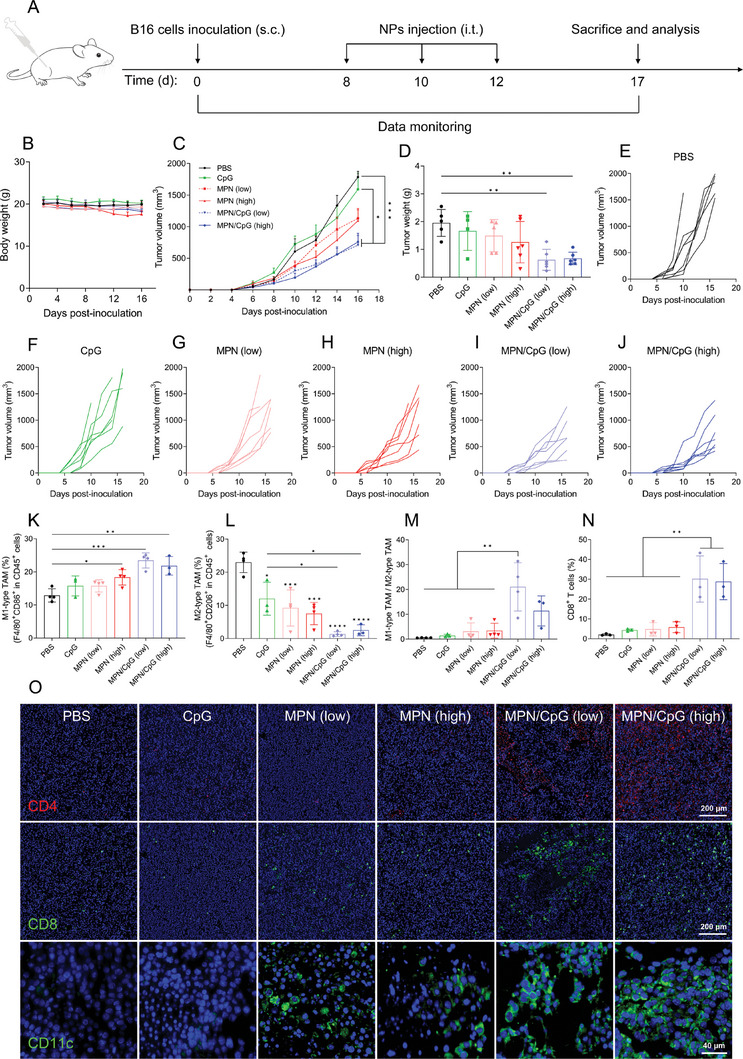
Local delivery of MPN/CpG inhibited the tumor growth and remodeled the tumor immune environment. A) Schematic diagram of the tumor treatment plan, where i.t. represents intratumoral injection. B) The weight of mice in each treatment group over the treatment period (n = 7). Tumor growth curves C) and tumor weight D) after different treatments. E–J) Tumor growth curves of individual mouse in different treatment groups (n = 7). K–M) Flow cytometry analysis of M1‐type and M2‐type macrophages in tumor tissues (n = 4). N) Flow cytometry analysis of CD8^+^ T cells in tumor tissues (n = 3). O) Immunofluorescence staining of tumor tissue slices. Nuclei: blue, CD4: red, CD8: green, CD11c: green. Scale bar = 40 or 200 µm. Data are presented as means ± SD or means ± SEM. Significance symbols without additional annotation represent significant comparisons with the control group. The statistical significance between the two groups was calculated by Student's t‐test, where *^#^P* < 0.05 was considered statistically significant. More than two groups were calculated by one‐way ANOVA with Tukey's *post hoc* test. *P < 0.05, **P < 0.01, ***P < 0.001, ****P < 0.0001.

To evaluate the therapeutic effect of MPN/CpG, mice received subcutaneous inoculation of B16 melanoma and subsequent intratumoral injection of MPN/CpG, MPN, CpG according to the schedule depicted in **Figure** **5**A. The mouse weight and tumor growth were monitored. No significant change in mouse body weight for all treatments over the experiment period (Figure 5B), and H&E staining of major organs did not reveal noticeable damage (Figure S8, Supporting Information), indicating that all treatments did not induce apparent toxicity. The tumor growth was significantly inhibited by the MPN/CpG treatment while the MPN treatment showed only moderate inhibition, and the CpG treatment had no significant impact on tumor growth. Surprisingly, the increased concentrations of Mn^2+^ in MPN/CpG exhibited similar tumor inhibition effects compared to low concentrations (Figure 5C,E–J). After isolation of the tumors, the average tumor weight of the MPN/CpG (low) and MPN/CpG (high) treatment groups was only one‐third of that in the PBS treatment group, suggesting that MPN/CpG was effective in controlling tumor growth (Figure 5D). Subsequent histological analysis of tumor staining revealed that in both the MPN/CpG (low) and MPN/CpG (high) treatment groups, there was a significant reduction in the nuclear density and nuclear staining of tumor cells, as indicated by H&E staining. Furthermore, bright green fluorescence was observed in TUNEL staining (Figure S9, Supporting Information), further confirming the effective inhibition of tumor cells. Compared to MPN/CpG, free Mn^2+^ + CpG was less effective in controlling tumor growth (Figure S10B–E, Supporting Information). Flow cytometry analysis revealed that the levels of granzyme B‐positive CD8^+^ T cells (Figure S10F,G, Supporting Information) and CD8^+^ T cells (Figure S10H, Supporting Information) in the blood were significantly higher in the MPN/CpG treatment group than in the Mn^2+^ + CpG treatment group. This indicates that local delivery of MPN/CpG can activate systemic immunity more effectively than a mixture of Mn^2+^ + CpG.

We next explored the immune microenvironment in the tumor by detecting the population of macrophages, T cells and DCs using flow cytometry. It was observed that treatment with MPN/CpG upregulated the CD86 expression and downregulated CD206 expression on the surface of F4/80^+^ macrophages, thereby significantly elevated the CD86^+^/CD206^+^ ratio. The results suggested that MPN/CpG treatment could reprogram the immunosuppressive M2 phenotype of tumor‐associated macrophages into an immunostimulatory M1 phenotype. Specifically, the percentage of M1‐type TAMs in the tumor microenvironment increased to 23.45% and 21.83% in the MPN/CpG (low) and MPN/CpG (high) treatment groups (Figure 5K), while the percentage of M2‐type TAMs decreased to 1.35% and 2.5% (Figure 5L). The M1/M2 ratio also significantly increased, reaching 37.3 and 20.2 times that of the PBS group (Figure 5M). Moreover, the number of tumor‐infiltrating CD8 T cells in the MPN/CpG (low) and MPN/CpG (high) treatment groups was significantly higher than in other treatment groups, reaching 30.2% and 28.9% (Figure 5N). This result was further confirmed by immunofluorescence staining of tumor tissue sections, which revealed the obviously enhanced fluorescence intensity of infiltrating CD4, CD8 T cells, and DCs (Figure 5O), compared to the PBS group. Together, the results suggest that MPN/CpG exhibits a potent tumor therapeutic effect and can remodel the tumor microenvironment, transforming a “cold” tumor into a “hot” tumor.

### 2.6 MPN/CpG Readily Migrate to Lymph Nodes In Vivo

**Figure 6:**
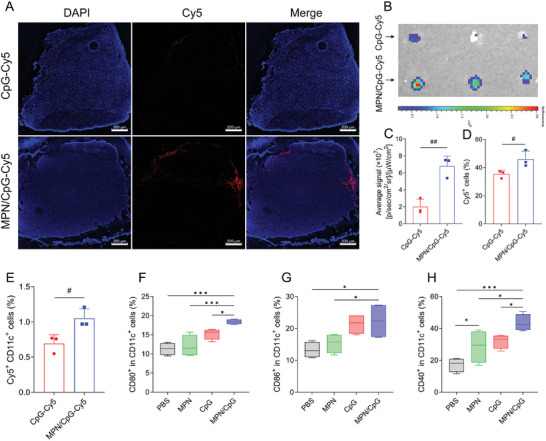
Migration of MPN/CpG to lymph nodes and DC activation in lymph nodes. A) Frozen sections of popliteal lymph nodes at 24 hours after footpad injection in mice, with cell nuclei stained using DAPI (blue), scale bar = 200 µm. Fluorescence images B) and quantification of the fluorescence signal C) of popliteal lymph nodes 24 hours after footpad injection of CpG‐Cy5 or MPN/CpG‐Cy5 in mice. D, E) Flow cytometry analysis of cellular uptake of CpG‐Cy5 or MPN/CpG‐Cy5 by cells or CD11c‐positive cells in lymph node. F) CD80, G) CD86 and H) CD40 expression on CD11c^+^ DCs in popliteal lymph nodes which were detected by flow cytometry at 12 hours after footpad injection (n = 4). Data are presented as means ± SD. The statistical significance between the two groups was calculated by Student's t‐test, where *^#^P* < 0.05 was considered statistically significant. More than two groups were calculated by one‐way ANOVA with Tukey's *post hoc* test. *P < 0.05, **P < 0.01, ***P < 0.001.

Adjuvants are recognized as key components in vaccines that can drive innate and adaptive anti‐tumor immunity. Lymph node is the main place where DCs are activated, uptake antigen and present antigen to T cells. We next explored the potential of MPN/CpG to migrate to lymph nodes and activate DCs. To assess the migration of MPN/CpG to lymph nodes, we subcutaneously injected Cy5‐labeled free CpG and MPN/CpG into the footpads of mice. After 24 hours, mouse popliteal lymph nodes were isolated and processed into frozen sections for observation. As shown in **Figure** **6**A, the MPN/CpG‐Cy5 group exhibited a clear and bright red fluorescence signal, primarily distributed in the peripheral cortex of the lymph nodes, which may be associated with the higher presence of lymphatic vessels in the outer region of the lymph nodes. In contrast, the CpG‐Cy5 group showed only faint fluorescence signals. Fluorescence imaging of popliteal lymph nodes using a small animal in vivo imaging system (IVIS) revealed that the average fluorescence signal of MPN/CpG‐Cy5 was 3.38 times higher than that of CpG‐Cy5 (Figure 6B,C). Furthermore, the number of Cy5‐positive cells in the lymph nodes of the MPN/CpG‐Cy5 group was significantly higher than that in the CpG‐Cy5 group (Figure 6D). These results indicating that compared to free CpG, MPN/CpG has a stronger ability to migrate to lymph nodes. Subsequently, the uptake of MPN/CpG by dendritic cells within lymph nodes was assessed by flow cytometry. The results showed that the MPN/CpG‐Cy5 group had the highest level of Cy5^+^CD11c^+^ cells (Figure 6E), indicating the efficient uptake of MPN/CpG‐Cy5 by dendritic cells.

Based on these results, we hypothesized that after the migration of MPN/CpG to the lymph nodes and uptake by DCs, it could effectively activate DCs. To validate this hypothesis, isolated popliteal lymph nodes were made into cell suspensions for flow cytometry analysis. Compared to the other groups, the MPN/CpG group exhibited the highest expression levels of CD80, CD86, and CD40 (Figure 6F–H), indicating a high level of DC activation. Taken together, the nanoadjuvant MPN/CpG can effectively migrate to lymph nodes in vivo, be taken up by dendritic cells, and subsequently promote the maturation of DCs within the lymph nodes.

### 2.7 MPN/CpG Drives Antigen‐Specific Cellular and Humoral Immune Response In Vivo

**Figure 7:**
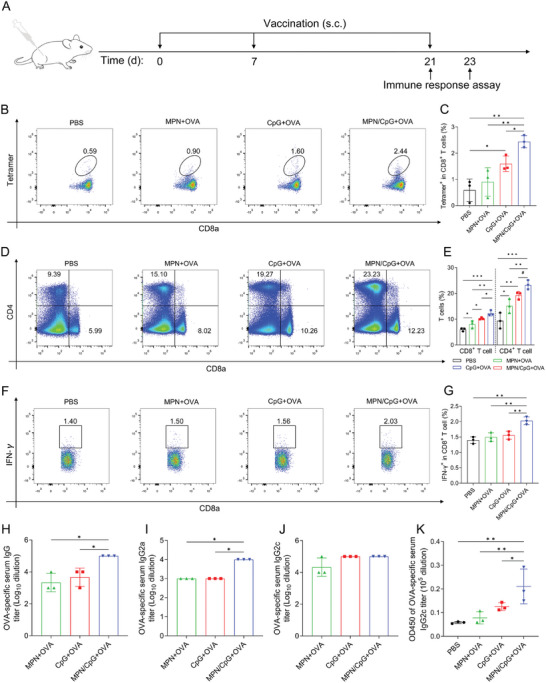
MPN/CpG activates specific cellular and humoral immunity in vivo. A) Vaccination schedule, where s.c. represents subcutaneous injection. B, C) Flow cytometric analysis of OVA‐specific CD8^+^ T cell levels in the blood at day 7 after the second vaccination (n = 3). D, E) The levels of CD4^+^ T, CD8^+^ T, and (F, G) IFN‐γ^+^CD8^+^ T cells in the spleen were detected by flow cytometry on day 2 following the final vaccination (n = 3). H–K) ELISA analysis of serum levels of OVA‐specific IgG, IgG2a, IgG2c, and IgG1 on day 2 after the final vaccination (n = 3). Data are presented as means ± SD. Statistical significance was calculated by one‐way ANOVA with Tukey's *post hoc* test with *P < 0.05, **P < 0.01, ***P < 0.001, or by Student's t‐test with *^#^P* < 0.05.

In light of the excellent performance of the MPN/CpG nanoadjuvant in targeting lymph nodes and activating DCs, we sought to explore its potential as a cancer vaccine. Ovalbumin (OVA) was chosen as the model tumor antigen, and together with MPN/CpG formed a tumor vaccine. In vivo adaptive immune response including cellular and humoral immunity were assayed after vaccination of C57BL/6 mice according to the schedule shown in **Figure** **7**A. First, we evaluated the level of OVA‐specific T cells in the blood. The results demonstrated that the proportion of OVA Tetramer^+^CD8^+^ T cells in the MPN/CpG + OVA treatment group was significantly higher than in other treatment groups, indicating the most robust activation of OVA‐specific CD8^+^ T cell immunity (Figure 7B,C). Subsequently, we assessed the activation of T cells in the spleen. Compared to other treatment groups, both CD4^+^ T and CD8^+^ T cells were significantly increased in the MPN/CpG + OVA treatment group (Figure 7D,E). After secondary stimulation of spleen cells with OVA, the proportion of IFN‐γ^+^CD8^+^ T cells in the MPN/CpG + OVA treatment group remained significantly higher than in other treatment groups (Figure 7F,G). These results suggest that MPN/CpG, as a tumor vaccine, can effectively activate antigen‐specific T cell immune responses in vivo.

Furthermore, we evaluated the levels of OVA‐specific IgG and its subtypes, IgG1, IgG2a, and IgG2c, in the serum after vaccine immunization. IgG1 antibodies are typically associated with Th2‐type responses, while IgG2a and IgG2c antibodies are considered to reflect Th1‐type immune levels.^[^ ^34^ ^]^ The results indicated that the OVA‐specific IgG and IgG2a antibody titers in the MPN/CpG + OVA treatment group (Figure 7H,I) and the absorbance values at the highest titers (Figure S12A,B, Supporting Information) were significantly higher than in the other two groups. Although the IgG2c antibody titers were the same as the CpG + OVA treatment group, the absorbance values at the highest titers were still significantly higher than in the CpG + OVA treatment group (Figure 7J,K). In contrast, IgG1, associated with Th2‐type immune responses, showed no significant increase in either antibody titers or the highest titers of absorbance values compared to the other treatment groups (Figure S12C,D, Supporting Information). This suggests that tumor vaccine (MPN/CpG + OVA) induced antigen‐specific humoral immunity is highly correlated with Th1‐type immune responses. In conclusion, MPN/CpG combined with OVA antigen can trigger strong antigen‐specific cellular and humoral immune responses in vivo.

### 2.8 MPN/CpG Effectively Prevents Tumor Growth in the Prophylactic Tumor Model

**Figure 8:**
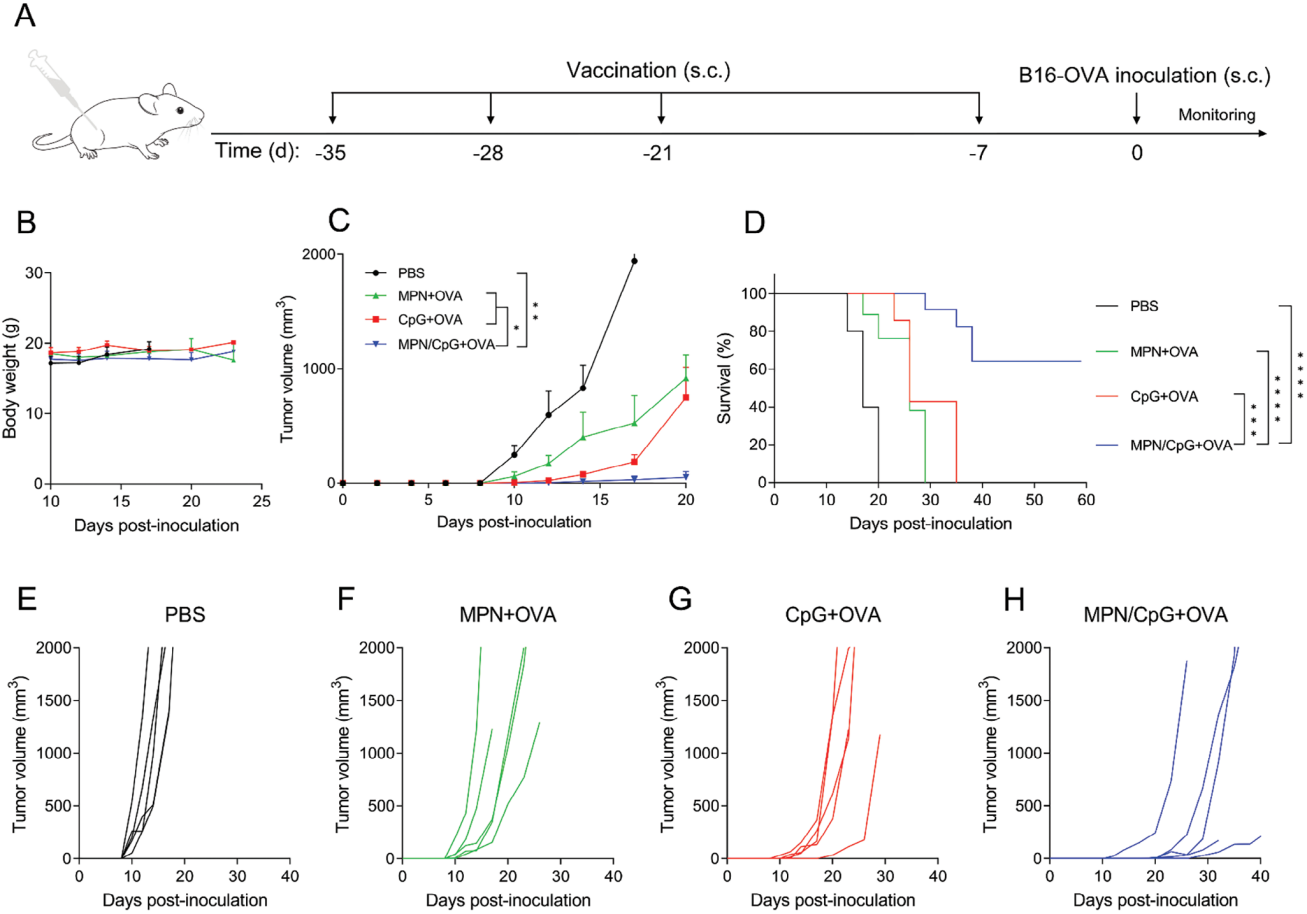
The anti‐tumor effect of MPN/CpG nanoadjvuant in the B16‐OVA tumor bearing prophylactic model. A) Vaccination schedule, with s.c. indicating subcutaneous injection. B) Changes in body weight of mice in each group after B16‐OVA tumor inoculation (n = 5). C) Average tumor growth curves (n = 5) and D) survival curves of mice after the mice received different vaccination and subsequent B16‐OVA tumor challenge (n = 5). E–H) Individual tumor growth curves for mice in different treatment groups (n = 5). Data are presented as means ± SEM. Statistical significance was calculated by one‐way ANOVA with Tukey's *post hoc* test. *P < 0.05, **P < 0.01, ***P < 0.001, ****P < 0.0001. Survival curves were compared by the Log‐rank test.

Following confirmation that the tumor vaccine, consisting of MPN/CpG and OVA, effectively activates specific cellular and humoral immune response, we assessed its preventive effect on B16‐OVA melanoma. The vaccination strategy is illustrated in **Figure** **8**A, where, seven days after the final vaccination, all mice were injected with B16‐OVA cells, and tumor growth was monitored. Throughout the entire treatment period, there was no significant change in the body weight of mice in all treatment groups, indicating the safety of the vaccine (Figure 8B). Tumor growth curves revealed that the PBS treatment group failed to effectively inhibit tumor growth, while the MPN + OVA and CpG + OVA treatment groups exhibited moderate inhibition. In contrast, the MPN/CpG + OVA treatment group effectively controlled the growth of B16‐OVA tumors (Figure 8C), and the survival rate of mice was significantly increased (Figure 8D). After 30 days of tumor inoculation, almost all mice in the other treatment groups had died (Figure 8E–G), whereas the MPN/CpG + OVA treatment group had only one death, and one mouse even survived for more than 60 days (Figure 8H). In summary, the MPN/CpG vaccine loaded with the OVA antigen demonstrated significant efficacy in tumor prevention, simultaneously inhibiting tumor growth and extending the survival time of mice.

## 3 Discussion

Adjuvants are indispensable components in vaccines. When co‐administered with vaccine antigens, they can enhance the adaptive immune response and induce effective immune memory. Therefore, adjuvants are considered one of the crucial biotechnological solutions in vaccine development.^[^ ^35^ ^]^ With the rise of nanomedicine‐based metalloimmunotherapy, the potential of metal ions as immunomodulators has been gradually explored.^[^ ^36^ ^]^ For instance, hydroxyapatite rich in Ca^2+^ can eliminate macrophage tolerance to endotoxins.^[^ ^37^ ^]^ Co‐administration with CpG can reverse immune tolerance during Toll‐like receptor 9 (TLR9) signal activation,^[^ ^38^ ^]^ and concurrent use with TLR4 agonists can stimulate macrophages toward an M1 polarization phenotype.^[^ ^39^ ^]^ Especially, the most extensively studied Mn^2+^ has been recognized as an effective anti‐tumor agent or adjuvant. In murine models, intranasal, intravenous, or intratumoral injection of Mn^2+^ consistently induces a robust immune response.^[^ ^27^ ^]^ The addition of Mn^2+^ effectively enhances the efficacy of tumor vaccines, exhibiting excellent anti‐tumor effects across various tumor models.^[^ ^40^, ^41^, ^42^ ^]^ Additionally, reports suggest a synergistic interaction between Mn^2+^ and STING agonists, with Mn^2+^ significantly enhancing the type I IFN activity of STING agonists.^[^ ^43^ ^]^ Both nucleic acid and non‐nucleic acid STING agonists, when forming nanoparticles with Mn^2+^, synergistically inhibit tumor development.^[^ ^44^, ^45^, ^46^ ^]^ Therefore, exploring the synergistic interactions between Mn^2+^ and other types of adjuvants, especially TLR agonists, is crucial for expanding the scope of metalloimmunotherapy approaches.

In this study, a synergistic effect was observed between Mn^2+^ and the TLR9 agonist CpG. Their combined use significantly stimulated DCs maturation. Interestingly, even Mn^2+^ alone treatment induced high‐level expression of maturation markers, suggesting the potential of Mn^2+^ as an immunological adjuvant. This aligns with previous research findings.^[^ ^29^, ^47^ ^]^ However, free Mn^2+^ and CpG face challenges in effectively migrating to the lymph nodes. Lymph nodes, as important peripheral immune organs, harbor a variety of immune cells and serve as crucial sites for generating immune responses, especially playing a pivotal role in the suppression and clearance of tumors.^[^ ^48^ ^]^ In contrast, nanoparticles are more prone to migration to the lymph nodes. After subcutaneous injection, nanoparticles typically migrate to lymph nodes via two pathways based on their own size. One involves active transport facilitated by antigen‐presenting cells (APCs) in the skin that uptake the nanoparticles, and the other involves passive transport through lymphatic drainage.^[^ ^49^, ^50^ ^]^ Therefore, we utilized EGCG to self‐assemble Mn^2+^ and CpG into nanoadjuvant MPN/CpG. We verified the loading of CpG onto MPN by the fluorescence quenching effect of Cy5‐labeled CpG. This is because when fluorescent groups are densely packed on the surface of nanoparticles, energy and electron transfer leads to an increase in non‐radiative relaxation of the excited state, resulting in effective fluorescence quenching.^[^ ^51^, ^52^ ^]^ The in vivo results indicate that MPN/CpG can effectively migrate to the lymph nodes and upon uptake by DCs, subsequently strongly stimulate DC maturation. When MPN/CpG is combined with the OVA antigen to form a tumor vaccine, the vaccine induces a strong specific cellular and humoral immune response, showing the highest activation of OVA‐specific T cells, IFN‐γ^+^CD8^+^ T cells, and IgG antibody secretion. In tumor prevention experiments, the MPN/CpG vaccine, compared to other treatment groups, significantly inhibits tumor growth and prolongs the survival time of mice. However, in vitro experiments found that Mn^2+^ effectively stimulated BMDC maturation, but MPN did not have a significant effect. Previous studies have indicated that treatment with 100 µM EGCG in the presence of LPS impaired the expression of the co‐stimulatory molecules CD80 and CD86.^[^ ^53^, ^54^ ^]^ Therefore, it is possible that the results were due to the different stimulatory effect of EGCG on immune cells. It should be emphasized that this effect is limited to in vitro cell experiments. In our in vivo studies, MPN effectively stimulated the expression of co‐stimulatory molecules of DCs in lymph nodes, particularly CD40.

The synergistic effect of Mn^2+^ and CpG is also evident in the regulation of macrophage polarization. Macrophages, highly heterogeneous immune cells, can be polarized into the M1‐type, inhibiting tumor growth, or the M2‐type, promoting tumor growth, in the complex tumor microenvironment. However, in the tumor microenvironment, tumor‐associated macrophages predominantly exhibit the M2 type, creating a conducive environment for tumor growth and metastasis.^[^ ^55^ ^]^ Therefore, an increasing amount of research is focusing on regulating the polarization of tumor‐associated macrophages. Existing studies indicate that nanomaterials based on Mn^2+^ or CpG can induce the repolarization of M2‐type tumor‐associated macrophages to the M1‐type, effectively improving the immunosuppressive tumor microenvironment and inhibiting tumor growth.^[^ ^21^, ^30^, ^56^, ^57^ ^]^ In this study, MPN/CpG was found to enhance the repolarization of M2‐type macrophages to M1‐type by in vitro experiments. Interestingly, the repolarization effect of MPN/CpG was slightly weaker than that of free CpG, possibly due to the time needed for MPN/CpG to release CpG after cellular uptake, while free CpG can rapidly exert its function upon entering endosomes. In in vivo experiments, the ability of free CpG to regulate macrophage polarization decreased rapidly in tumors due to its susceptibility to degradation and difficulty in uptake by immune cells. Free CpG only moderately decreased M2‐type TAMs and increased M1‐type TAMs in tumors, which was merely comparable to the level of MPN regulation. In comparison, the repolarization effect of MPN/CpG is more pronounced, with a significant increase in the ratio of M1 to M2 TAMs. However, this synergistic effect appears to be insensitive to the concentration of Mn^2+^, as even when the Mn^2+^ concentration in MPN/CpG is increased tenfold, the repolarization effect does not significantly improve. One possible explanation is that there is an upper limit to the cellular Mn^2+^ content, and to maintain Mn^2+^ homeostasis, cells use transport proteins such as SLC30A10 and Ferroportin (FPN) to expel excess Mn^2+^ from the cell.^[^ ^58^ ^]^ Therefore, high concentrations of Mn^2+^ may not sufficiently activate intracellular pathways to exert their functions. Furthermore, the therapeutic effect of MPN/CpG seems not only due to the activation of immune cells. Live/dead staining experiments revealed that both MPN and MPN/CpG could induce a certain level of cell death in tumor cells. With prolonged exposure, the degree of cell death further intensified (Figure S11, Supporting Information).

Although we observed a synergistic effect of Mn^2+^ and CpG, the specific mechanism remains unknown. This study reveals that Mn^2+^ and CpG can synergistically promote the secretion of type I interferons. Type I IFNs play a crucial role in regulating protective immunity, such as promoting dendritic cell maturation, enhancing B cell antibody production, and improving T cell and natural killer cell function.^[^ ^59^ ^]^ Previous research has unveiled two mechanisms by which Mn^2+^ induces type I IFN production. One mechanism involves the activation of the STING–TBK1–IRF3 signaling pathway, wherein Mn^2+^ induces TBK1 and p65 phosphorylation. In the presence of a STING agonist, phosphorylated TBK1 further induces IRF3 phosphorylation, leading to the cascade amplification of the STING signal and the production of type I IFNs.^[^ ^26^, ^43^ ^]^ Another mechanism, similar to the activation mode of Toll‐like receptor 4 (TLR4) agonist lipopolysaccharide (LPS), involves interacting with membrane TLR4 to activate the TLR4–MyD88–NF‐κB/AP‐1 and TLR4–TRIF–IRF7 signaling pathways, resulting in type I IFN production.^[^ ^60^ ^]^ Therefore, we hypothesize that the coordinated action of Mn^2+^ and CpG regulates through similar signaling activation pathways. Our research indicates that MPN/CpG is initially internalized into lysosomes, followed by partial lysosomal escape, leading to the release of Mn^2+^ into the cytoplasm. A recent study by Han et al. reported that they synthesized a fluorinated assembly system (LFNPs) based on fluorinated EGCG (FEGCG). Their experiments revealed that LFNPs3‐3 could transfer from lysosomes to the cytoplasm, achieving lysosomal escape.^[^ ^61^ ^]^ Similarly, Wang et al. found that nanoliposomes synthesized from EGCG could achieve lysosomal escape through the proton sponge effect.^[^ ^62^ ^]^ An earlier study also confirmed that EGCG could increase lysosomal permeability.^[^ ^63^ ^]^ Based on these findings and our data, we speculate that MPN/CpG‐achieved lysosomal escape might be through an EGCG‐mediated proton sponge effect. Therefore, this escape mechanism is likely to simultaneously activate the TLR9 pathway within endosomes, and the STING–TBK1–IRF3 pathway in the cytoplasm, thereby cascading the amplification of type I interferon production. Subsequently, we experimentally validated the above hypothesis. The colocalization of MPN/CpG and TLR9 fluorescence suggested a potential interaction between them. Western blot analysis confirmed that MPN/CpG could activate the STING‐TBK1 and TLR9‐NF‐κB pathways, inducing phosphorylation of STING, TBK1, IRF3 and p65. This indicates that MPN/CpG primarily functions through the activation of both the TLR9 and STING pathways.

## 4 Conclusion

In summary, the study demonstrated the potential of Mn^2+^ as an immune modulator and its synergistic effects with CpG. Through successful coordination with EGCG, we have developed a dual‐adjuvant nanoplatform, MPN/CpG. This nanoadjuvant exhibits robust synergistic effects both in vitro and in vivo by activating the STING/TLR9‐NF‐κB pathways. Compared to free adjuvants, the MPN/CpG nanoadjuvant is more readily taken up by dendritic cells and accumulates in lymph nodes. Intratumoral injection can significantly improve the immunosuppressive tumor microenvironment, induce repolarization of M2‐type tumor‐associated macrophages to M1‐type, and enhance T cell and dendritic cells infiltration in the tumor. As a tumor vaccine adjuvant, MPN/CpG induces potent specific T cell immune responses, effectively preventing tumor growth. Therefore, our strategy of combining metal ions with TLR agonists using a polyphenol provides a promising avenue for metalloimmunotherapy.

## 5 Experimental Section

### Materials

Dulbecco's modified eagle medium (DMEM), RPMI 1640 medium, PBS buffer, fetal bovine serum (FBS), and penicillin‐streptomycin solution were purchased from Gibco or BDBIO HangZhou China. MgCl_2_‐6H_2_O, CaCl_2_, MnCl_2_‐4H_2_O, CuCl_2_‐2H_2_O, ZnCl_2_, AlCl_3_‐6H_2_O, and FeCl_3_‐6H_2_O were purchased from Kelong Chemical Co., Ltd. Epigallocatechin gallate (EGCG) was purchased from Adamas. Ovalbumin (OVA) was purchased from Sigma‐Aldrich. CpG (5′‐T*C*C*A*T*G*A*C*G*T*T*C*C*T*G*A*C*G*T*T‐3′), CpG‐Cy5, and qRT‐PCR primers were synthesized by Sangon Biotech. Pam3CSK4 and LPS were purchased from InvivoGen. Cell Counting Kit‐8 (CCK‐8) was purchased from Abbkine Scientific Co., Ltd. Mouse IL‐6 ELISA kit was purchased from BioLengd, TNF‐α ELISA kit was purchased from FineTest, and IFN‐β ELISA kit was purchased from Rexin Biotech. Goat anti‐mouse antibodies IgG (HRP), IgG1 (HRP), IgG2a (HRP), and IgG2c (HRP) were purchased from Abcam. Flow cytometry mouse antibodies FITC‐CD11c, APC‐CD80, PE‐CD40, PerCP/Cyanine5.5‐CD86, APC‐Cy7‐CD45, APC‐F4/80, PE‐CD3 were purchased from BioLegend, FITC‐CD8a (KT15) was purchased from Abcam. PE‐H‐2K^b^ OVA‐Tetramer was purchased from MBL. FITC‐CD8a, PE‐CD4, PE‐CD206, Cytofix/Cytoperm Plus were purchased from BD Biosciences. Type I collagenase purchased from Absin. In vivo melanoma models were established using healthy female C57BL/6 mice (Chengdu Dashuo Laboratory Animal Center, China). All the animal experiments were approved by the local Ethic Committee of Sichuan University (Chengdu, China) and performed according to the animal use protocol of Sichuan University (K2023024).

### Preparation and Characterization of MPN/CpG Nanoparticles

MnCl_2_‐4H_2_O (86 µL, 5 mg mL^−1^) and EGCG (25 µL, 10 mg mL^−1^) were added to a vial containing 1.5 mL of ultrapure water. The mixture was stirred at room temperature for 10 minutes. It was then centrifuged at 2000 rpm for 10 minutes using a 100 kDa ultrafiltration tube to remove excess material and obtain MPN nanoparticles. Subsequently, the 5 µg solution of CpG was introduced to the MPN solution and allowed to stand for 30 minutes at room temperature to complete the formation of MPN/CpG.

The average size and zeta potential of MPN/CpG nanoparticles were measured using dynamic light scattering (DLS) (Zetasizer Nano ZS90, Malvern). Nanoparticle morphology was observed using transmission electron microscopy (TEM, Tecnai G2 F20 S‐TWIN, FEI). The binding of MPN to CpG was detected by agarose gel electrophoresis and fluorescence spectrophotometry.

### In Vitro Evaluation of the Ability of Metal Ions to Stimulate IL‐6 Secretion

RAW264.7 cells of 1 × 10^5^ were seeded in 48‐well cell culture plates and incubated at 37 °C for 24 hours. Different concentrations (125 µM, 250 µM, 500 µM, 1000 µM) of metal ions (MgCl_2_·6H_2_O, CaCl_2_, MnCl_2_·4H_2_O, CuCl_2_·2H_2_O, ZnCl_2_, AlCl_3_·6H_2_O, FeCl_3_·6H_2_O) and CpG (250 ng mL^−1^) were added and then incubated at 37 °C for 24 hours. Cell supernatants were collected for IL‐6 ELISA.

RAW264.7 cells of 5 × 10^4^ were seeded in 48‐well cell culture plates and incubated at 37 °C for 24 hours. Different concentrations of Pam3CSK4, LPS, CpG (250 ng mL^−1^, 500 ng mL^−1^, 1000 ng mL^−1^) and MgCl_2_·6H_2_O (125 µM) added and then incubated at 37 °C for 24 hours. Cell supernatants were collected for IL‐6 ELISA.

BMDCs of 1 × 10^6^ were seeded in 12‐well cell culture plates and cultured at 37 °C for 24 hours. Different concentrations (7.8125 µM, 15.625 µM, 31.25 µM, 62.5 µM, 125 µM, 250 µM, 500 µM, and 1000 µM) of MnCl_2_·4H_2_O and CpG (500 ng mL^−1^) were added respectively and cultured for 24 hours at 37 °C, and the cell supernatants were collected for IL‐6 ELISA.

### Cell Viability Assay

Cell viability was assessed using the Cell Counting Kit‐8 (CCK‐8) assay. RAW264.7 cells were seeded at a density of 3 × 10^5^ cells per well in a 48‐well plate and cultured overnight. After treatment with different materials, the cells were incubated at 37 °C for 24 hours. Fresh culture medium was then replaced, and 1% CCK‐8 reagent was added. The cells were further incubated at 37 °C in the dark for 30 minutes. Subsequently, the absorbance at 450 nm was measured using a microplate reader (BioTek, Synergy H1), and the results were recorded.

### Bone Marrow‐Derived Dendritic Cells (BMDCs) Extraction

Female C57BL/6 mice aged 6 to 8 weeks were euthanized, and their femurs and tibiae were separated. The bone marrow cavity was flushed with PBS buffer to obtain bone marrow cells. These cells were then passed through a 70 µm cell sieve to remove impurities. The supernatant was separated by centrifugation at 1200 rpm for 5 minutes. A 1 mL volume of red blood cell lysis buffer was added, and the lysate was left at room temperature for 5 minutes. The lysis reaction was terminated by adding 9 mL of PBS buffer. After centrifugation, the cell precipitate was resuspended in R10 medium, which consists of RPMI 1640 medium, 10% FBS, 1% PS, and 50 µM β‐ME. The cell concentration was adjusted to 1 × 10^6^ cells mL^−1^. The resuspended cells were plated into a 6‐well cell culture seed with 3 mL in each well. GM‐CSF (20 ng mL^−1^) and IL‐4 (10 ng mL^−1^) were added, and this day was recorded as day 0. On day 3, the cell supernatant was completely replaced with R10 medium containing GM‐CSF and IL‐4. On day 5, 3 mL of R10 medium containing GM‐CSF and IL‐4 was added to each well. Loose adherent BMDCs could be collected on day 7.

### MPN/CpG Cellular Uptake In Vitro

BMDCs of 5 × 10^5^ were seeded in 35 mm glass‐bottomed confocal Petri dishes and placed in a cell culture incubator for overnight culture. CpG‐Cy5 and MPN/CpG‐Cy5 were separately added to BMDCs and co‐cultured at 37 °C. The cells were washed with PBS buffer and replaced with fresh RPMI 1640 medium. Then, 50 nM Lyso‐Tracker Green (Beyotime) or PE‐CD289 (TLR9) were added and incubated at 37 °C for 1 hour. Subsequently, the cells were washed with PBS buffer and replaced with fresh RPMI 1640 medium. Hoechst 33 342 (Beyotime) was added and stained for 10 minutes, followed by washing with PBS buffer. Finally, the cells were photographed using confocal laser scanning microscope (CLSM, LSM880, Zeiss).

### Analysis of BMDCs Activation and Cytokine Secretion

BMDCs of 1 × 10^6^ were seeded in 12‐well plates for overnight culture, and PBS, CpG (1 µg mL^−1^), MnCl_2_‐4H_2_O (125 µM), MPN, MPN/CpG were added to culture for 24 hours, respectively, and centrifugation was performed at 1200 rpm for 5 minutes, and the cell supernatants were taken to test for IL‐6, TNF‐α, and IFN‐β using ELISA kits. Cellular precipitates were washed twice with FACS buffer (1% BSA + PBS), incubated with CD16/32 antibodies for 10 min at room temperature, and incubated with PerCP/Cyanine5.5‐CD86, APC‐CD80, PE‐CD40, and FITC‐CD11c antibodies for 30 minutes on ice, and washed with FACS buffer and detected by flow cytometry (FACS Canto II, BD).

### qRT‐PCR Analysis

BMDCs of 1 × 10^6^ were seeded to 12‐well plates for overnight incubation and treated with various materials for 24 hours. Afterward, the cells were washed twice with PBS buffer, and total RNA was extracted using an RNA Extraction Kit (TIANGEN). The extracted RNA was reverse‐transcribed into cDNA using a Reverse Transcription Kit (abm). Subsequently, real‐time fluorescent quantitative PCR was performed with 2 × qPCR MasterMix (abm) and primers on a PCR detection system (CFX960, Bio‐Rad). The resulting data were used to calculate relative expression levels using the 2^−∆∆t^ method. Below were the primer sequences used. (**Table** **1**).

**Table 1:** Primer sequences used for qRT‐PCR.

Gene	Primer sequences
*Il‐6*	F: 5′‐GAGGATACCACTCCCAACAGACC‐3′ R: 5′‐AAGTGCATCATCGTTGTTCATACA‐3′
*Tnfa*	F: 5′‐TGGGAGTAGACAAGGTACAACCC‐3′ R: 5′‐CATCTTCTCAAAATTCGAGTGACAA‐3′
*Ifnb*	F: 5′‐TGGGAGATGTCCTCAACTGC‐3′ R: 5′‐CCAGGCGTAGCTGTTGTACT‐3′
*Gapdh*	F: 5′‐TGGTGAAGGTCGGTGTGAAC‐3′ R: 5′‐CCATGTAGTTGAGGTCAATGAAGG‐3′

### Macrophage Polarization Assay

The RAW264.7 cells were seeded at a density of 5 × 10^5^ cells per well in a 6‐well plate and cultured overnight. After incubation with 100 ng mL^−1^ LPS for 48 hours to induce differentiation, the cells were treated with different materials in fresh DMEM culture medium and incubated at 37 °C for 24 hours. The old culture medium was removed, and the cells were washed twice with flow cytometry staining buffer (1% BSA + PBS). After incubating with CD16/32 antibody at room temperature for 10 minutes, the cells were stained on ice for 30 minutes with FITC‐CD86 and PE‐CD206 antibodies. Following washing with flow cytometry staining buffer, the cells were analyzed using a flow cytometer (FACS Canto II, BD).

### Western Blot Analysis

BMDCs 1 × 10^6^ were seeded in a six‐well plate and cultured overnight. After treatment with different materials for 0.5 hours, the cells were lysed with cell lysis buffer and centrifuged at 12,000 g for 5 minutes to collect the supernatant. 5 × SDS‐PAGE loading buffer was added and the mixture was denatured at 100 °C for 5 minutes. Equal protein loading was ensured, and the samples were loaded onto a 10% SDS‐PAGE gel. Electrophoresis was performed at 150 V for 40 minutes. The proteins were then transferred to a PVDF membrane at 400 mA for 40 minutes on ice. The membrane was blocked with rapid blocking buffer (Yeasen) at room temperature for 10–15 minutes, followed by three washes with 1 × TBST. The membrane was incubated with primary antibodies, including STING (ABclonal), P‐STING (Immunoway), TBK1 (ABclonal), P‐TBK1 (ABmart), IRF3 (HUABIO), P‐IRF3 (CST), p65 (CST), P‐p65 (CST), and GAPDH (Proteintech), for 1 hour at room temperature or overnight at 4 °C. After three washes with 1 × TBST, the membrane was incubated with secondary antibodies for 1 hour at room temperature, followed by three additional washes with 1 × TBST. Chemiluminescence detection and analysis were then performed.

### Lymph Node Targeting Analysis

In vivo, C57BL/6 mice received subcutaneous injections of CpG‐Cy5 and MPN/CpG‐Cy5 into their footpads. After 24 hours, popliteal lymph nodes were harvested, and the fluorescence intensity was measured using an in vivo imaging system (IVIS Spectrum, PerkinElmer). Furthermore, lymph nodes were mechanically disrupted through a 70 µm cell sieve to obtain cell suspensions. These cell suspensions were stained with CD16/32 and FITC‐CD11c flow cytometry antibodies to analyze the in vivo uptake of dendritic cells (DCs). To observe the specific localization of CpG‐Cy5 and MPN/CpG‐Cy5 within the lymph nodes, lymph nodes were cryosectioned into 10 µm slices. These sections were fixed with 4% paraformaldehyde for 10 minutes, washed with PBS buffer, and then incubated with 50 µl of anti‐fade mounting medium containing DAPI (Beyotime). The sections were observed under a confocal laser scanning microscope (CLSM, LSM880, Zeiss).

For DC activation, C57BL/6 mice received subcutaneous injections of CpG‐Cy5 and MPN/CpG‐Cy5 nanoparticles into their footpads. After 12 hours, popliteal lymph nodes were harvested. The lymph nodes were mechanically disrupted through a 70 µm cell sieve to obtain cell suspensions. These cell suspensions were then incubated with CD16/32 antibodies for 10 minutes, followed by staining with FITC‐CD11c, PerCP/Cyanine5.5‐CD86, APC‐CD80, and PE‐CD40 antibodies for 30 minutes. Subsequently, analyzed by flow cytometry (FACS Canto II, BD).

### Analysis of In Vivo Tumor Therapeutic Efficacy

Each C57BL/6 mouse was subcutaneously injected with 5 × 10^5^ B16 cells on the posterior side. The mouse's weight and tumor volume were measured every two days, and the tumor volume was calculated using the formula: Volume = 0.5 × length × width^2^. When the tumor volume reached 100–200 mm^3^, intratumoral injection of respective materials was administered. On the seventeenth day after tumor inoculation, mice were euthanized, and tumor tissues and major organs were isolated. Tumor tissues were digested with a solution (containing 1 mg mL^−1^ collagenase I and 200 µg mL^−1^ DNase I) at 37 °C for 30 minutes. The resulting cell suspension was filtered through a 70 µm cell strainer for flow cytometry analysis. After incubation with CD16/32 antibody for 10 minutes, cells were stained with APC‐Cy7‐CD45, APC‐F4/80, FITC‐CD86, and PE‐CD206 antibodies for 30 minutes to analyze M1‐type (CD45^+^F4/80^+^CD86^+^) and M2‐type (CD45^+^F4/80^+^CD206^+^) macrophages. APC‐Cy7‐CD45, PE‐CD3, and FITC‐CD8 antibodies were used for 30 minutes to analyze CD8 T cells (CD45^+^CD3^+^CD8^+^).

The cells were seeded in 24‐well cell culture plates at a concentration of 1 × 10^7^ cells per well, stimulated with Trp2 (30 µg mL^−1^) for 1.5 hours at 37 °C, followed by incubation with 1 × brefeldin A for another 4 hours. The cells were subsequently stained with CD16/32 antibody for 10 minutes. The cells were then stained with APC‐Cy7‐CD45, PE‐CD3, and FITC‐CD8 antibodies for 30 minutes, washed twice with FACS buffer. The cells were then fixed and permeabilized with 250 µL of Fixation/Permeabilization solution (BD Biosciences) for 20 minutes at 4 °C. After washing twice with 1 × BD Perm/Wash buffer, the cells were stained overnight at 4 °C with BV421‐Granzyme B antibody. After washing twice with 1 × BD Perm/Wash buffer, perform detection using a flow cytometer (FACS Canto II, BD). Other organs were prepared as slices for immunohistochemical analysis.

### In Vivo Immune Response Analysis

C57BL/6 mice were randomly divided into four groups: PBS, CpG + OVA, MPN + OVA, and MPN/CpG + OVA, and the vaccines were injected subcutaneously on days 0, 7, and 21 (OVA dosage: 100 µg mouse^−1^, CpG dosage: 5 µg mouse^−1^). Seven days after the second vaccination, the blood of mice was collected, and 1 mL of red blood cell lysis buffer (Beyotime) was added to complete lysis of red blood cells, which were then co‐incubated with CD16/32, FITC‐CD8a (KT15), and PE‐H‐2K^b^ OVA‐Tetramer antibodies, and flow cytometry assay was performed to analyze the activation of OVA‐specific CD8^+^ T cells.

Mouse blood and spleens were collected two days after the third inoculation. The blood was centrifuged at 2500 rpm for 10 minutes, and the upper serum was tested for IgG, IgG1, IgG2a, and IgG2c antibody secretion using ELISA kits. Mouse spleens were mechanically disrupted through a 70 µm cell sieve, and red blood cells were completely lysed by adding red blood cell lysis buffer (Beyotime). Some of the splenic lymphocytes were isolated and co‐incubated with FITC‐CD8a and PE‐CD4 antibodies for the analysis of CD4^+^ and CD8^+^ T cells. Splenic lymphocytes were then seeded in 24‐well cell culture plates at a concentration of 1 × 10^7^ cells per well, stimulated with OVA (100 µg mL^−1^) for 1.5 hours at 37 °C, followed by incubation with 1 × brefeldin A for another 4 hours. The cells were subsequently stained with CD16/32 antibody for 10 minutes and FITC‐CD8a antibody for 30 minutes, washed twice with FACS buffer, fixed and permeabilized with 250 µL of Fixation/Permeabilization solution (BD Biosciences) for 20 minutes at 4 °C. After washing twice with 1 × BD Perm/Wash buffer, the cells were stained overnight at 4 °C with PE‐IFNγ antibody. After washing twice with 1 × BD Perm/Wash buffer, perform detection using a flow cytometer (FACS Canto II, BD).

### Analysis of Tumor Prophylactic Effects In Vivo

C57BL/6 mice were randomly divided into four groups: PBS, CpG + OVA, MPN + OVA, and MPN/CpG + OVA. They were subjected to a total of four vaccinations with an interval of 7 days, except for the last dose, which was administered after a 14‐day interval. Each vaccination consisted of OVA (100 µg mouse^−1^) and CpG (5 µg mouse^−1^). Seven days after the final vaccination, 1 × 10^5^ B16‐OVA cells were subcutaneously injected into the rear side of each mouse. The mice's body weight and tumor volume were measured every two or three days. The tumor volume was calculated using the formula: Volume = 0.5 × length × width^2^. Mice were euthanized when their tumor volume exceeded 2000 mm^3^.

### Statistical Analysis

All data were shown as mean ± S.D. or mean ± S.E.M. The statistical significance between the two groups was calculated by Student's t‐test, where *^#^P* < 0.05 was considered statistically significant. More than two groups were calculated by one‐way ANOVA followed by Tukey's multiple comparisons test for *post hoc* tests. *P < 0.05, **P < 0.01, ***P < 0.001, ****P < 0.0001. The survival curve was analyzed by the Log‐rank test. GraphPad Prism 9.5.1 was used for statistical analyses.

## Conflict of Interest

The authors declare no conflict of interest.

## Author Contributions

Z.L. performed resources, methodology, data curation, formal analysis, investigation, software, wrote – the original draft. S.L. performed methodology, investigation. Y.X. performed methodology, investigation. X.L. performed methodology, investigation. B.Z. performed methodology, investigation. X.Z. performed funding acquisition, project administration. Q.Z. performed conceptualization, methodology, supervision, project administration, funding acquisition, wrote – the original draft, wrote – review & edited the original draft. Q.A. performed conceptualization, methodology, supervision, project administration, funding acquisition, wrote – the original draft, wrote – review & edited the original draft.

## Supporting information

Supporting Information

## Data Availability

Research data are not shared.
