# Supplementary material for: A Multi‐Functional Nanoadjuvant Coupling Manganese with Toll‐Like 9 Agonist Stimulates Potent Innate and Adaptive Anti‐Tumor Immunity
Source: Adv Sci (Weinh). 2024 Sep 11;11(41):2402678. doi: 10.1002/advs.202402678 (PMC11538688; doi:10.1002/advs.202402678)
Supplement: Supplementary file 1 — Supporting Information [file ADVS-11-2402678-s001.pdf]

ADVANCED SCIENCE
Open Access

Supporting Information

for *Adv. Sci.*, DOI 10.1002/advs.202402678

A Multi-Functional Nanoadjuvant Coupling Manganese with Toll-Like 9 Agonist Stimulates Potent Innate and Adaptive Anti-Tumor Immunity

Zhongjie Liu, Shu Li, Yang Xiao, Xiaoyang Liu, Bin Zhang, Qin Zeng, Qiang Ao* and Xingdong Zhang*

Supporting Information

A Multi-functional Nanoadjuvant Coupling Manganese with Toll-like 9 Agonist Stimulates Potent Innate and Adaptive Anti-tumor Immunity

Zhongjie Liu[1, 2], *Shu Li*[1], *Yang Xiao*[1], *Xiaoyang Liu*[3], *Bin Zhang*[1], *Qin Zeng*[1, 2, *], *Qiang Ao*[1, 2, *], *Xingdong Zhang*[1, 2]

[1] College of Biomedical Engineering, Sichuan University, Chengdu, 610064, China.

[2] NMPA Key Laboratory for Quality Research and Control of Tissue Regenerative Biomaterial & Institute of Regulatory Science for Medical Device & National Engineering Research Center for Biomaterials, Sichuan University, Chengdu, Sichuan, 610064, China.

[3] Orthopedic Research Institution, Department of Orthopedics, West China Hospital, Sichuan University, Chengdu 610041, China.

*Corresponding authors E-mail: Qin Zeng: qzeng8156@scu.edu.cn; Qiang Ao: aoqiang@scu.edu.cn; NO.29 Wangjiang Road, Chengdu, China, 610064.

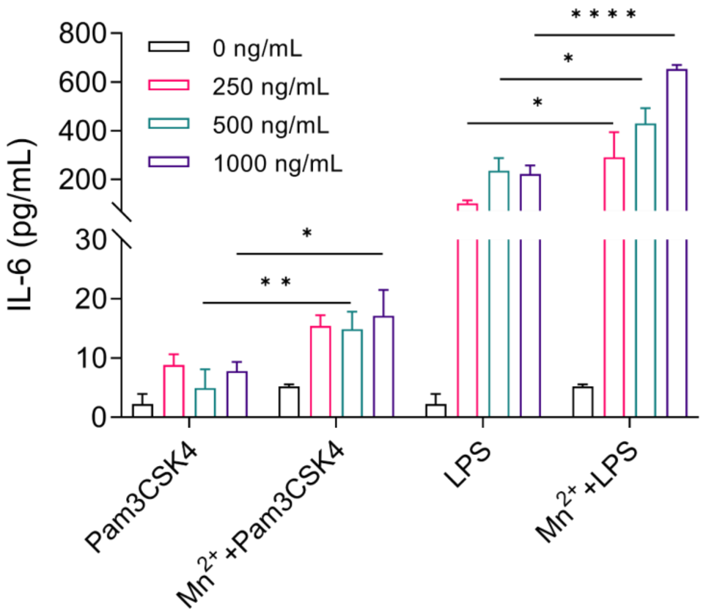

Figure S1. Measurement of IL-6 secretion after co-incubation of RAW264.7 cells with 125 μM Mn^{2+} and different concentrations of TLR agonists for 24 hours. Pam3CSK4: TLR2 agonist, LPS: TLR4 agonist. Data are presented as means ±

SD. Statistical significance was calculated by one-way ANOVA with Tukey's *post hoc* test. *$P < 0.05$, **$P < 0.01$, ***$P <$ 0.001, ****$P < 0.0001$.

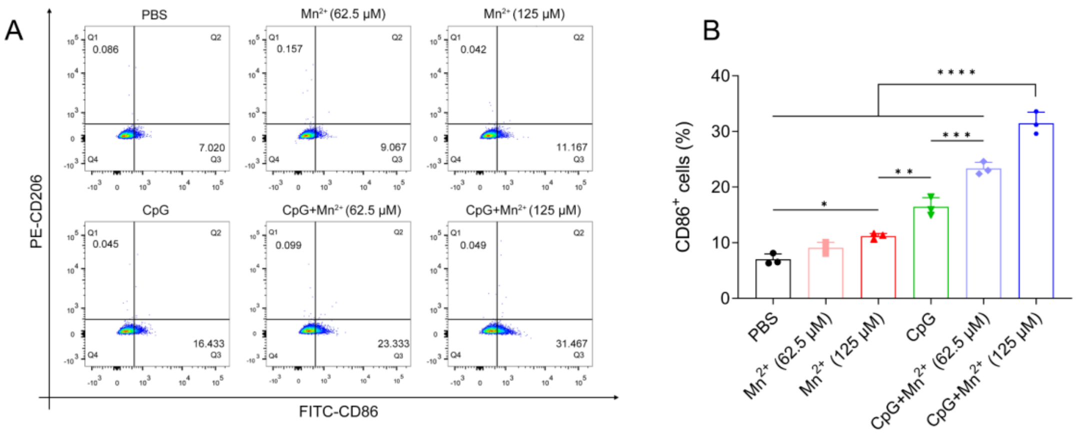

Figure S2. (A) Detection of M1-type ($CD86^+$) and M2-type ($CD206^+$) macrophages in RAW264.7 cells after different treatments using flow cytometry. (B) Quantitative analysis of $CD86^+$ cells. Data are presented as means ± SD. Statistical significance was calculated by one-way ANOVA with Tukey's *post hoc* test. *$P < 0.05$, **$P < 0.01$, ***$P < 0.001$, ****$P <$ 0.0001.

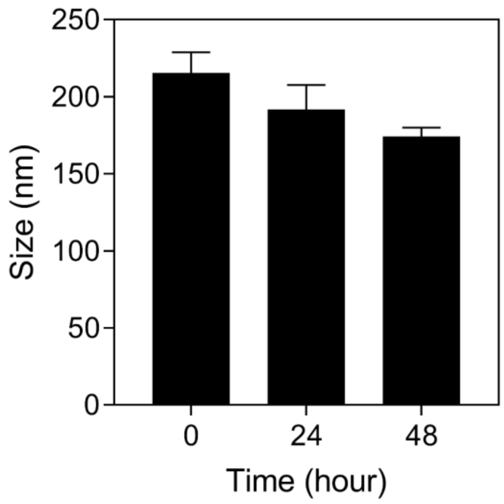

Figure S3. Dynamic light scattering (DLS) measures the average particle size of MPN/CpG in aqueous solution at different time points. Data are presented as means ± SD.

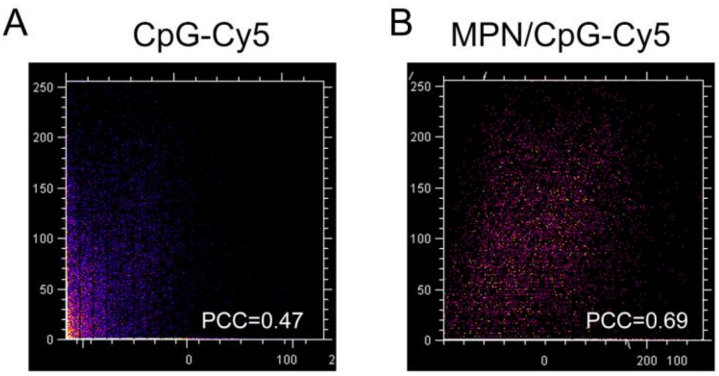

Figure S4. Analysis of the Pearson's correlation coefficient (PCC) using ImageJ after co-incubation of CpG-Cy5 and MPN/CpG-Cy5 with BMDCs for 0.5 hours.

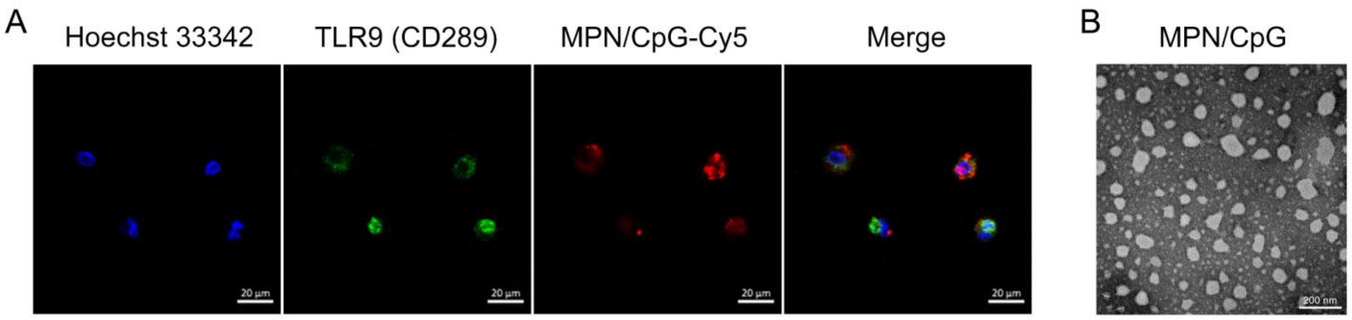

Figure S5. (A) Intracellular colocalization of TLR9 and MPN/CpG nanoparticles was detected by confocal laser scanning microscopy (CLSM) after BMDCs were treated with MPN/CpG-Cy5 for 0.5 hours., scale bar = 20 μm. The cell nucleus and TLR9 were stained with Hoechst 33342 (blue) and PE-CD289 (green), respectively. (B) TEM image of MPN/CpG after 4 hours in a solution at pH 5.1, scale bar = 200 nm.

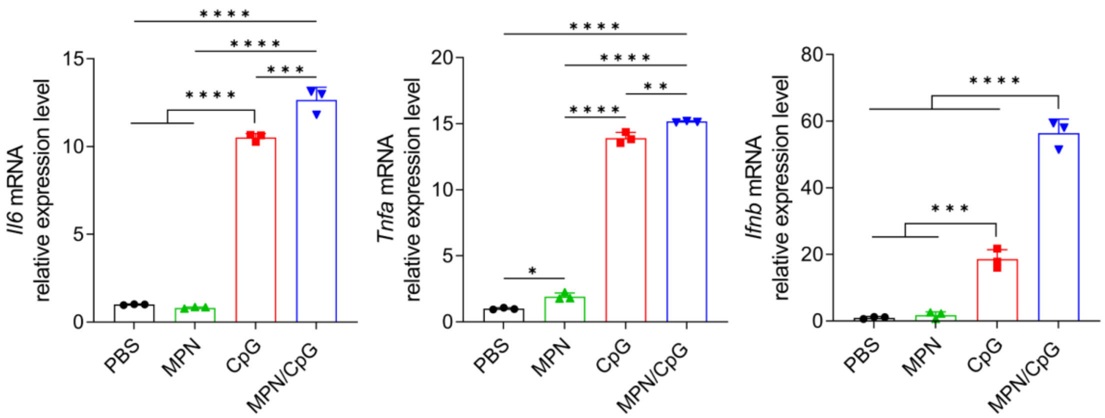

Figure S6. Relative expression levels of *Il6*, *Tnfa*, and *Ifnb* mRNA in BMDCs after different treatments were detected by qRT-PCR. Data are presented as means ± SD. Statistical significance was calculated by one-way ANOVA with Tukey's *post hoc* test. *$P < 0.05$, **$P < 0.01$, ***$P < 0.001$, ****$P < 0.0001$.

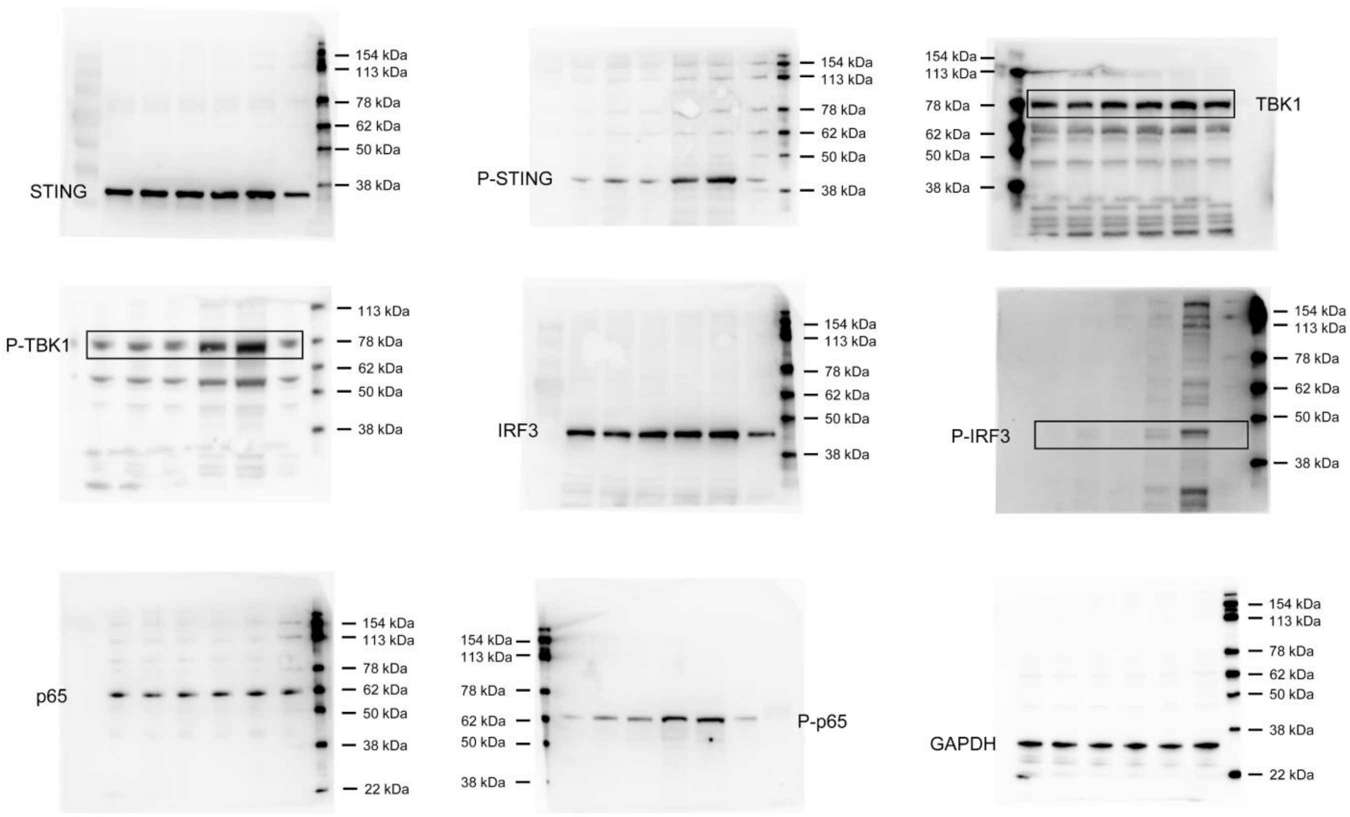

Figure S7. The original, unedited scanned image of the western blot for Figure 4F.

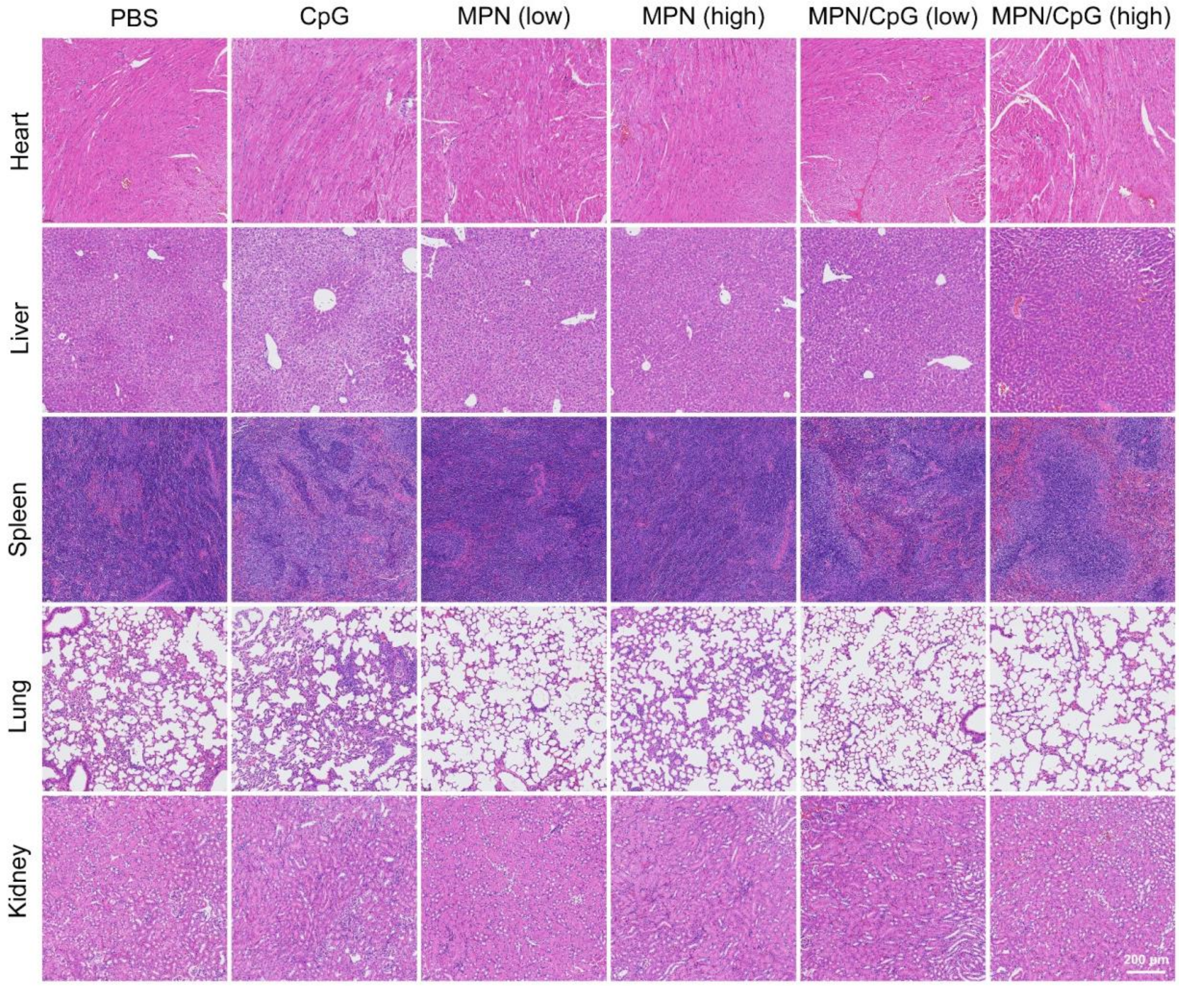

Figure S8. H&E staining of heart, liver, spleen, lung, and kidney tissue sections from different treatment groups, scale bar = 200 μm.

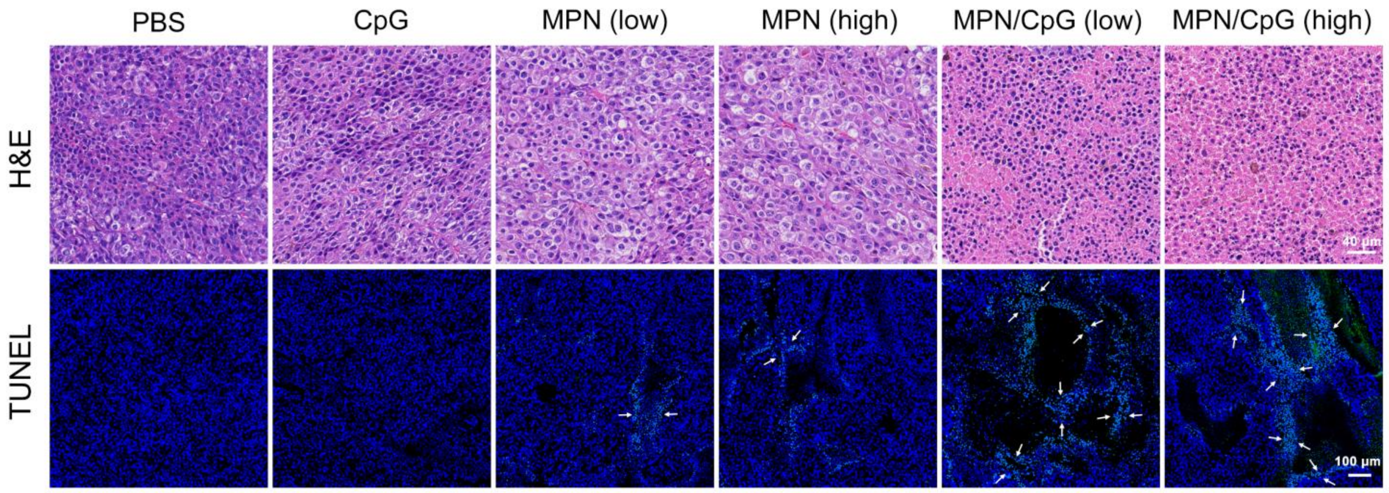

Figure S9. H&E and TUNEL staining of tumor tissue sections from different treatment groups. The cerulean blue areas indicated by the arrows represent TUNEL-positive staining. scale bar = 40 or 100 μm.

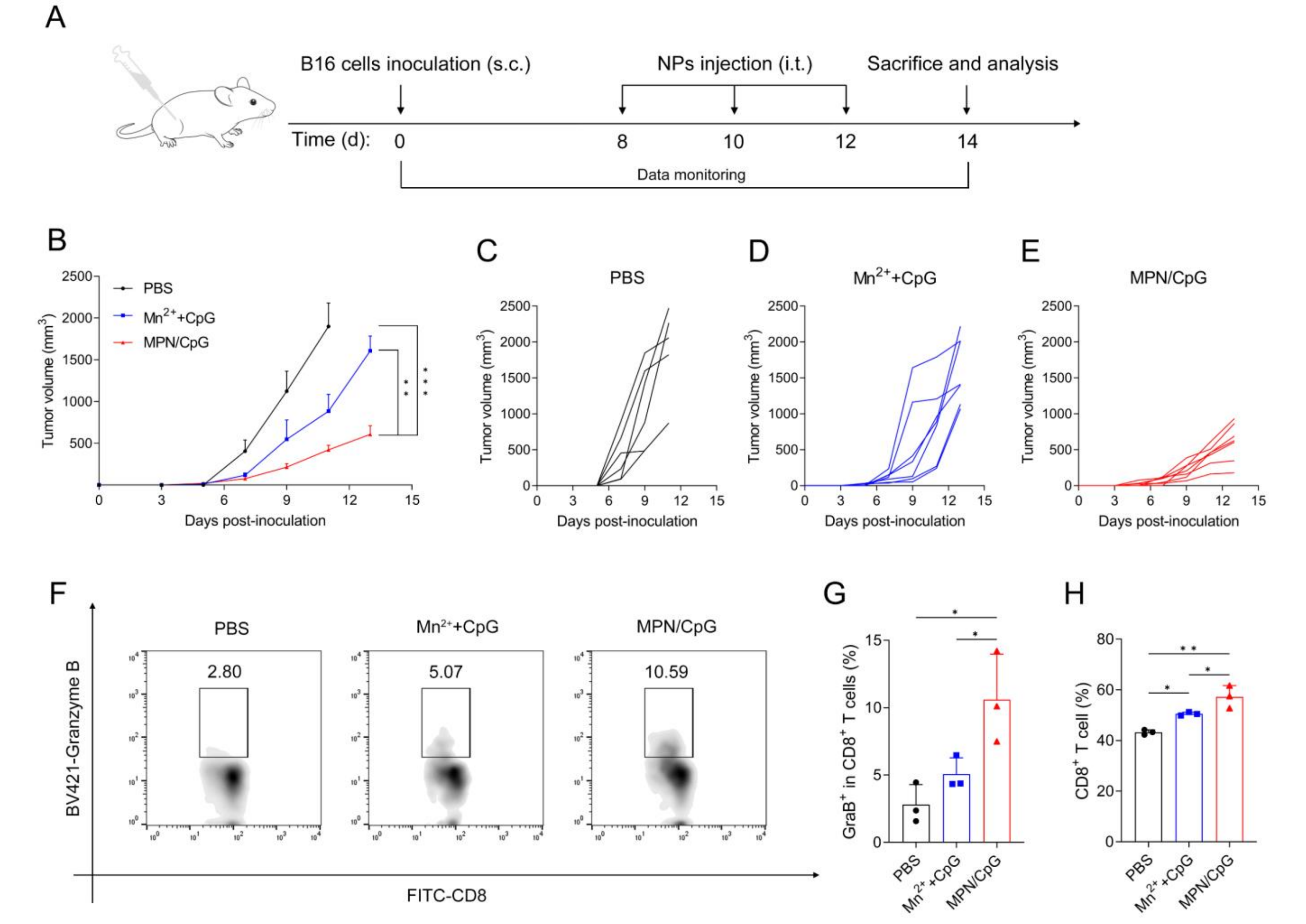

Figure S10. The local therapeutic effect of immune adjuvant in B16 tumor-bearing mouse model. (A) Schematic diagram of

the tumor treatment plan, where i.t. represents intratumoral injection. (B) Average tumor growth curve and (C-E) individual tumor growth curves after different treatments. Data are presented as means ± SEM. (F-G) Flow cytometry analysis of the percentages of granzyme B^+ in $CD8^+$ T cells and (H) $CD8^+$ T cells in blood after different treatments. Data are presented as means ± SD. Statistical significance was calculated by one-way ANOVA with Tukey's *post hoc* test with $^*P < 0.05$, $^{**}P < 0.01$.

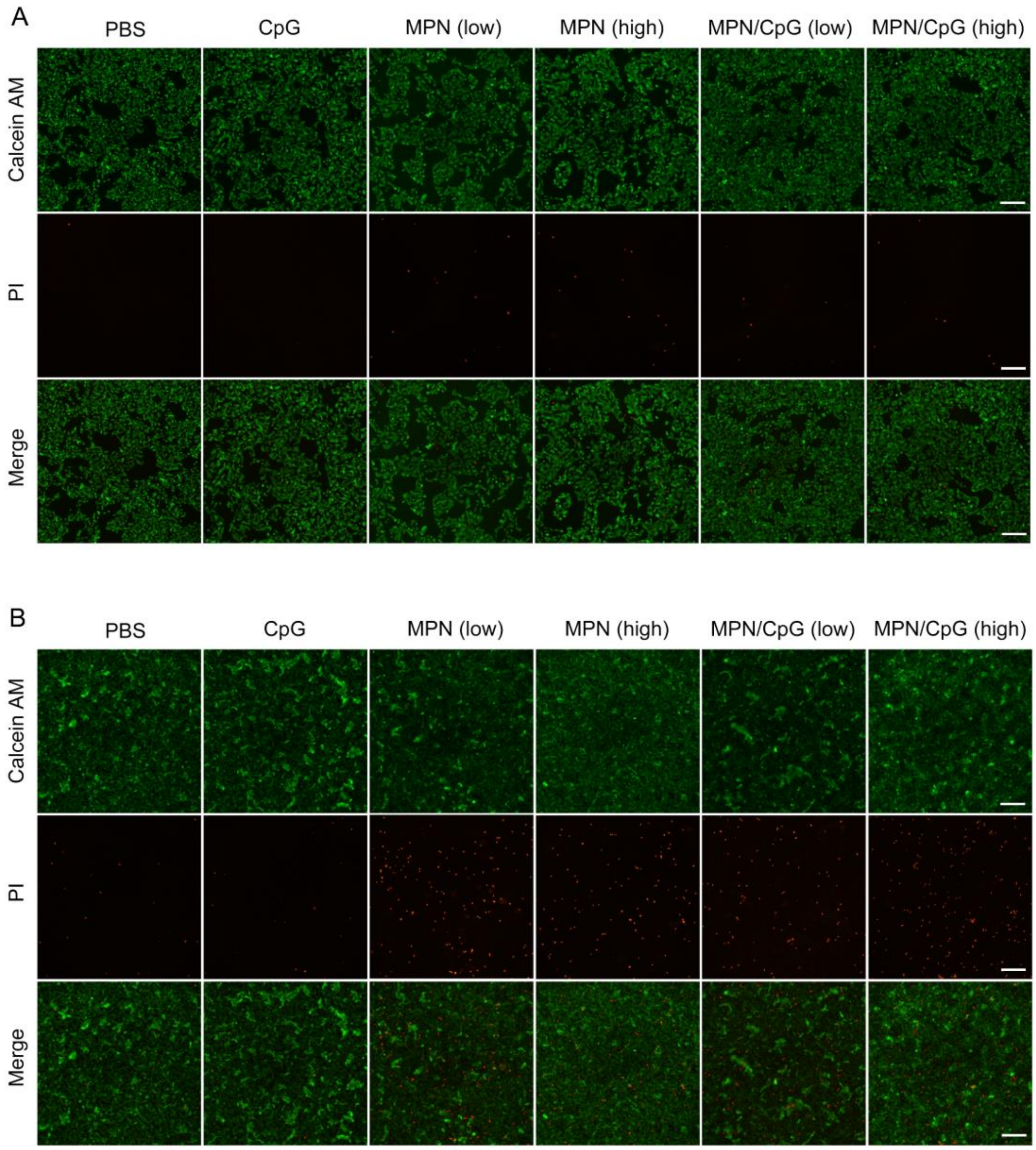

Figure S11. B16 cells treated differently were subjected to live/dead cell double staining at (A) 24 hours and (B) 72 hours, with Calcein-AM in green and PI in red. Scale bar = 2 mm.

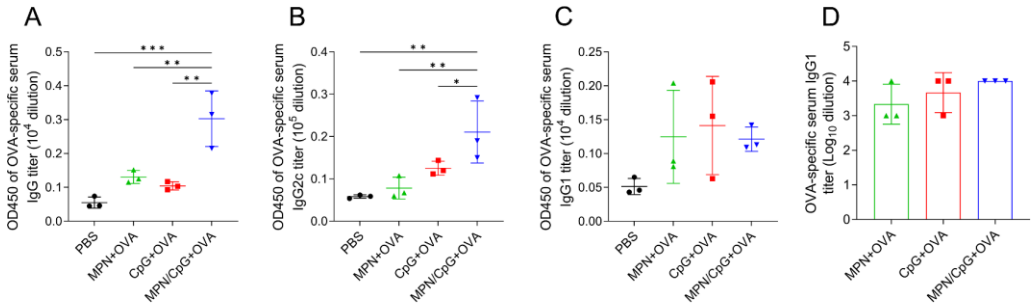

Figure S12. On the 23rd day of treatment, the highest antibody titers of OVA-specific (A) IgG, (B) IgG2c, and (C) IgG1 in mouse serum were measured by absorbance (n = 3). (D) OVA-specific IgG1 antibody titers (n = 3). Data are presented as means ± SD. Statistical significance was calculated by one-way ANOVA with Tukey's *post hoc* test. *$P < 0.05$, **$P < 0.01$, ***$P < 0.001$, ****$P < 0.0001$.
